# LAB-1 Targets PP1 and Restricts Aurora B Kinase upon Entrance into Meiosis to Promote Sister Chromatid Cohesion

**DOI:** 10.1371/journal.pbio.1001378

**Published:** 2012-08-21

**Authors:** Yonatan B. Tzur, Carlos Egydio de Carvalho, Saravanapriah Nadarajan, Ivo Van Bostelen, Yanjie Gu, Diana S. Chu, Iain M. Cheeseman, Monica P. Colaiácovo

**Affiliations:** 1 Department of Genetics, Harvard Medical School, Boston, Massachusetts, United States of America; 2 Department of Biology, San Francisco State University, San Francisco, California, United States of America; 3 Whitehead Institute for Biomedical Research, and Department of Biology, Massachusetts Institute of Technology, Cambridge, Massachusetts, United States of America; Stowers Institute for Medical Research, United States of America

## Abstract

Successful execution of the meiotic program depends on the timely establishment and removal of sister chromatid cohesion. LAB-1 has been proposed to act in the latter by preventing the premature removal of the meiosis-specific cohesin REC-8 at metaphase I in *C. elegans*, yet the mechanism and scope of LAB-1 function remained unknown. Here we identify an unexpected earlier role for LAB-1 in promoting the establishment of sister chromatid cohesion in prophase I. LAB-1 and REC-8 are both required for the chromosomal association of the cohesin complex subunit SMC-3. Depletion of *lab-1* results in partial loss of sister chromatid cohesion in *rec-8* and *coh-4 coh-3* mutants and further enhanced chromatid dissociation in worms where all three kleisins are mutated. Moreover, *lab-1* depletion results in increased Aurora B kinase (AIR-2) signals in early prophase I nuclei, coupled with a parallel decrease in signals for the PP1 homolog, GSP-2. Finally, LAB-1 directly interacts with GSP-1 and GSP-2. We propose that LAB-1 targets the PP1 homologs to the chromatin at the onset of meiosis I, thereby antagonizing AIR-2 and cooperating with the cohesin complex to promote sister chromatid association and normal progression of the meiotic program.

## Abstract 2

At the onset of the first meiotic division, the protein LAB-1 recruits the PP1 phosphatase to cohesion complexes, preventing Aurora B kinase from targeting cohesins for degradation prematurely and thereby ensuring proper progression of meiotic events in *C. elegans*.

## Author Summary

A critical step for achieving successful cell division is the regulation of how the cohesin complexes that bind sister chromatids are initially deposited, then maintained, and finally removed to allow the chromatids to separate into daughter cells. This is particularly challenging during meiosis, when the sister chromatids must remain partially connected to each other through the first division. In organisms that have a single focal centromere on each chromosome, such as mammals and flies, cohesin is protected through the first meiotic division by the protein Shugoshin, which binds the PP2A phosphatase. PP2A counteracts phosphorylation by the Aurora B kinase; if certain cohesins are phosphorylated by Aurora B they become targeted for removal, which allows the chromatids to separate. In the nematode *C. elegans*, the chromosomes lack a localized centromere and the predicted Shugoshin homolog is not required for protection of cohesins; instead, this function is executed in metaphase of the first meiotic division by the protein LAB-1. But it is not completely understood what leads to the deposition of cohesin prior to entry into meiosis and to its maintenance throughout early meiosis I. In this study, we show that LAB-1 is also required for the loading and maintenance of the cohesin complex. LAB-1 ensures that the chromatids are not separated prematurely, and thus enables the proper progression of events through prophase I of meiosis. We propose that LAB-1 may act at the onset of meiosis in a manner akin to Shugoshin, by recruiting the PP1 phosphatase to counteract Aurora B kinase, thereby ensuring sister chromatid cohesion.

## Introduction

Timely establishment and subsequent removal of Sister Chromatid Cohesion (SCC) between sister chromatids is necessary to facilitate faithful segregation of chromosomes in mitosis and meiosis. Failure to correctly segregate chromosomes in either mitosis or meiosis has been associated with tumorigenesis, miscarriages, and congenital defects [1],[2]. Premature loss of SCC prevents the correct bipolar attachment of sister kinetochores to the mitotic spindle, whereas a delay in removing SCC may prevent segregation and therefore result in mitotic arrest and aneuploidy (reviewed in [3],[4]). During meiosis, the control of SCC establishment and removal is even more intricate, and many meiotic processes fail when SCC is compromised. In this specialized cell division program, one cycle of chromosome replication is followed by two consecutive rounds of chromosome segregation, thus reducing the chromosome number by half to produce haploid sperm and oocytes. At the onset of meiosis I, homologous chromosomes pair and undergo synapsis mediated by the formation of a proteinaceous scaffold called the synaptonemal complex (SC). When the SC disassembles, homologs remain attached to each other through chiasmata as a result of earlier crossover recombination events underpinned by flanking SCC. A tight regulation of the establishment of SCC is required for the normal progression of these meiotic events. However, while studies from a number of organisms have revealed key insights into the regulation of SCC removal, far less is known about how the establishment of SCC is regulated.

Cohesin is a highly conserved multisubunit complex that establishes SCC by binding the newly formed chromatid as it is synthesized. The four core subunits of cohesin are the structural maintenance of chromosomes proteins Smc1 and Smc3, the Scc1/Rad21 kleisin, and the accessory protein Scc3 (reviewed in [5],[6]). During meiosis in monocentric organisms such as flies, vertebrates, and yeast, dissolution of chromosome cohesion occurs in a stepwise manner and involves phosphorylation and degradation of Rec8, a meiosis-specific Scc1 paralog. First, cohesin is removed at the chromosome arms during meiosis I, but actively maintained at the pericentromeric regions until anaphase II segregation when the remaining cohesin subset is degraded [7]–[9]. At the end of meiosis I, members of the Shugoshin protein family prevent cohesin removal from the centromeres by recruiting protein phosphatase 2A (PP2A), which counteracts the phosphorylation of Rec8 by Aurora B Kinase, thereby sparing cohesin from separase-mediated degradation [10]–[19].

In *C. elegans*, the holocentric homolog pairs undergo a single crossover event located at the terminal thirds of chromosomes [20],[21]. Upon chromosome condensation, this off-center crossover leads to the characteristic cruciform shape of the bivalents, which display long and short arms that play an important role for correct alignment on the metaphase I plate [22],[23]. As in monocentric organisms, cohesin is also removed in a two-step process in *C. elegans*. While REC-8 is lost on the short arm in meiosis I, REC-8 is preserved on the long arm past homolog segregation. This process coincides with the differential loading of the Aurora B Kinase homolog AIR-2, which during diakinesis is observed exclusively on the short arms where it is proposed to phosphorylate REC-8, thus licensing its cleavage by Separase [24],[25]. Recently, two other kleisin homologs, COH-3 and COH-4, were found to also play a part in SCC during *C. elegans* meiosis, but their specific roles and localization are still unknown [26].

In both mammals and fission yeast two Shugoshin paralogs function in cohesin protection as well as in the spindle assembly checkpoint [14],[19],[27]–[29], yet it was suggested that the latter is the ancestral role of Shugoshin, and that the protection of sister chromatid cohesion evolved as its consequence [27]. So far only a single sequence-predicted Shugoshin homolog was identified in *C. elegans*, SGO-1, but neither AIR-2 localization nor sister chromatid association are compromised during meiosis I in *sgo-1* mutants [16],[30]. Instead, our studies suggested that the worm-specific LAB-1 (Long Arms of the Bivalent) protein participates in protecting REC-8 at the long arms during the metaphase I to anaphase I transition [30]. LAB-1 progressively forms continuous tracks throughout the full length of chromosomes starting at the onset of meiosis and co-localizes with the synaptonemal complex at pachytene [30]. During chromosome remodeling, LAB-1 becomes restricted to the long arms of the bivalents and, like Shugoshin in monocentric species, is finally removed from chromosomes in early anaphase I. *lab-1* hypomorphic mutants show a spreading of AIR-2 signals to both arms of the bivalents, similar to mutants of the protein phosphatase 1 (PP1) homolog *gsp-2*. These results are consistent with a model in which LAB-1 impacts sister chromatid cohesion in late meiosis I by regulating cohesin phosphorylation. Based on these and other observations, as well as the lack of evidence for a direct role of Shugoshin in protecting meiotic cohesin in *C. elegans*, we have speculated that due to the holocentric nature of *C. elegans* chromosomes, Shugoshin maintained its roles in the spindle attachment checkpoint, but LAB-1 evolved as part of a process to protect cohesin during meiosis in this organism [30]. However, both how and when PP1 function is directed and regulated remained open questions.

Here we describe an earlier and distinct role for LAB-1 in the establishment of SCC via PP1 regulation. Moreover, we demonstrate how failing to properly establish SCC influences various downstream meiotic events. Depletion of *lab-1* by RNAi reduces SCC and consequently impairs homolog pairing, alters the progression of meiotic recombination, and results in an increase in recombination intermediates (MSH-5 and ZHP-3 foci). We found that LAB-1, together with REC-8, is required for proper loading of SMC-3 and consequently for proper SC polymerization, which requires normal axis morphogenesis. While the different cohesin members and LAB-1 show some degree of interdependence with respect to either their initial localization or the subsequent maintenance of their localization on chromosomes, LAB-1 can promote partial SCC even in the absence of all three SCC-1 meiotic paralogs. Finally, underscoring a role in the regulation of phosphorylation, LAB-1 directly interacts with GSP-1 and its paralog GSP-2. Moreover, depletion of *lab-1* results in reduced GSP-2 and increased AIR-2 signals in early meiotic nuclei. We propose that LAB-1 specifically targets PP1 to chromosomes in early meiotic stages to antagonize AIR-2 phosphorylation and to promote sister chromatid cohesion, thus supporting the normal progression of downstream meiotic events.

## Results

### LAB-1 Depletion Reduces Pairing and Sister Chromatid Cohesion

**Figure 1:**
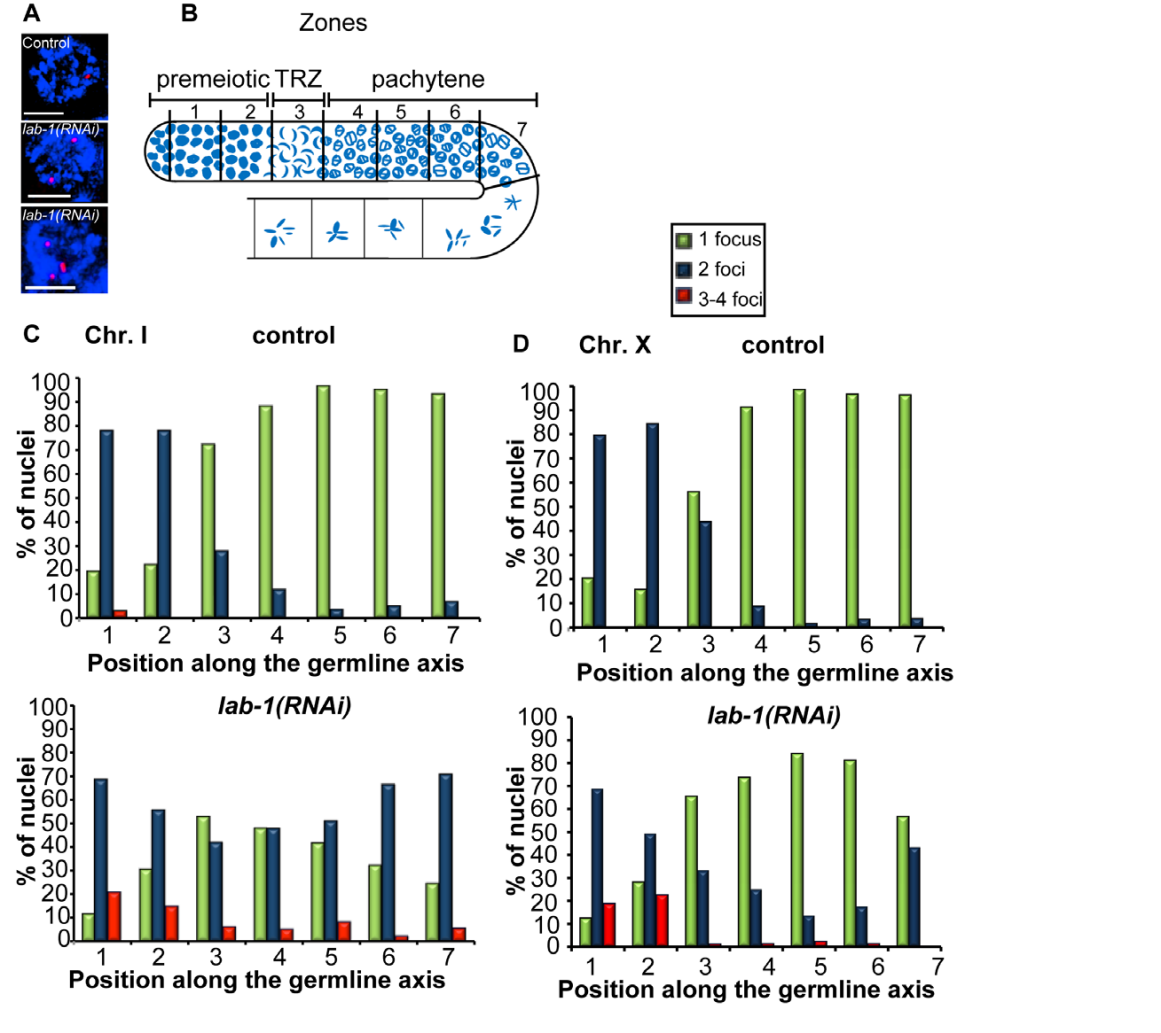
LAB-1 is required for homologous pairing. (A) High-magnification images of DAPI stained mid-pachytene nuclei (blue) hybridized with FISH probes targeting the pairing center region on chromosome I (red). Nuclei with either one, two, or three signals (foci) are depicted. Bars, 3 µM. (B) Diagram of a *C. elegans* germline indicating the position of the zones scored in the analysis of the progression of homologous pairing. (C, D) Graphs depicting the percentage of nuclei showing one, two, and three to four foci within each zone in control and *lab-1(RNAi)* gonads hybridized with FISH probes recognizing the chromosome I pairing center (C) and the X chromosome pairing center (D).

Our analysis of *lab-1(RNAi)* worms revealed the presence of >12 DAPI-stained bodies in 1.1% (*n* = 88) of oocytes at diakinesis, compared to the six DAPI-stained bodies corresponding to the six pairs of attached homologous chromosomes observed in wild type or the 7 to 12 univalents indicative of lack of chiasmata, suggesting instead a defect in sister chromatid cohesion (this study and [30]). To determine whether this defect results from a role for *lab-1* in early meiosis, we examined the effects of *lab-1* depletion on the various processes that take place earlier during prophase I (Figure S1). We first examined homologous chromosome pairing, a process that occurs upon entry into meiosis in most organisms (reviewed in [31]–[34]). To follow the progression of pairing in the germline, we used fluorescence in situ hybridization (FISH) with probes labeling the pairing center end of chromosome I. FISH signals either ≤0.75 µM or >0.75 µM apart represent paired and unpaired homologs, respectively (Figure 1A). In control gonads, homologous chromosomes are unpaired prior to meiotic entry and therefore only a few nuclei (*n* = 17/81) show a single FISH signal (Figure 1B and 1C, zones 1 and 2). Upon entry into meiotic prophase I (transition zone, which corresponds to the leptotene and zygotene stages; zone 3), the frequency of nuclei carrying a single focus increases (*n* = 52/72), and by pachytene, pairing is completed (Figure 1C, zones 4–7). In contrast, in *lab-1*-depleted gonads, pairing levels were reduced. Only 52% (*n* = 44/84) of nuclei in the transition zone carried paired homologs (Figure 1C, zones 3), and this was further decreased until only 24% (*n* = 9/37) of nuclei were observed with paired homologs at late pachytene (Figure 1C, zone 7, *p*<0.05, by the two-sided Fisher's Exact Test, 95% C.I.). Using a probe targeting the X chromosome pairing center revealed similar, albeit milder, results (Figure 1D). Although the reason for a stronger impairment of pairing on the autosomes compared to the X chromosome remains unclear, this has also been observed in other meiotic mutants [35]–[37]. Thus, depleting *lab-1* reduces homolog pairing, suggesting a previously unknown function for LAB-1 during prophase I.

In 20% (14/69) of premeiotic nuclei from *lab-1*-depleted gonads, we also noticed the presence of three or four FISH signals (Figure 1A), consistent with a defect in sister chromatid association (Figure 1C). At later stages, the frequency of nuclei with 3–4 signals decreased in *lab-1(RNAi)* gonads (5%, *n* = 2/37, at late pachytene). This temporal reduction could be explained either by residual LAB-1 or by a LAB-1-independent mechanism. Taken together, these observations suggest that LAB-1 may affect homologous pairing due to its role in the early establishment and/or maintenance of SCC.

### Meiotic DNA Double-Strand Break Repair Progression Requires LAB-1

**Figure 2:**
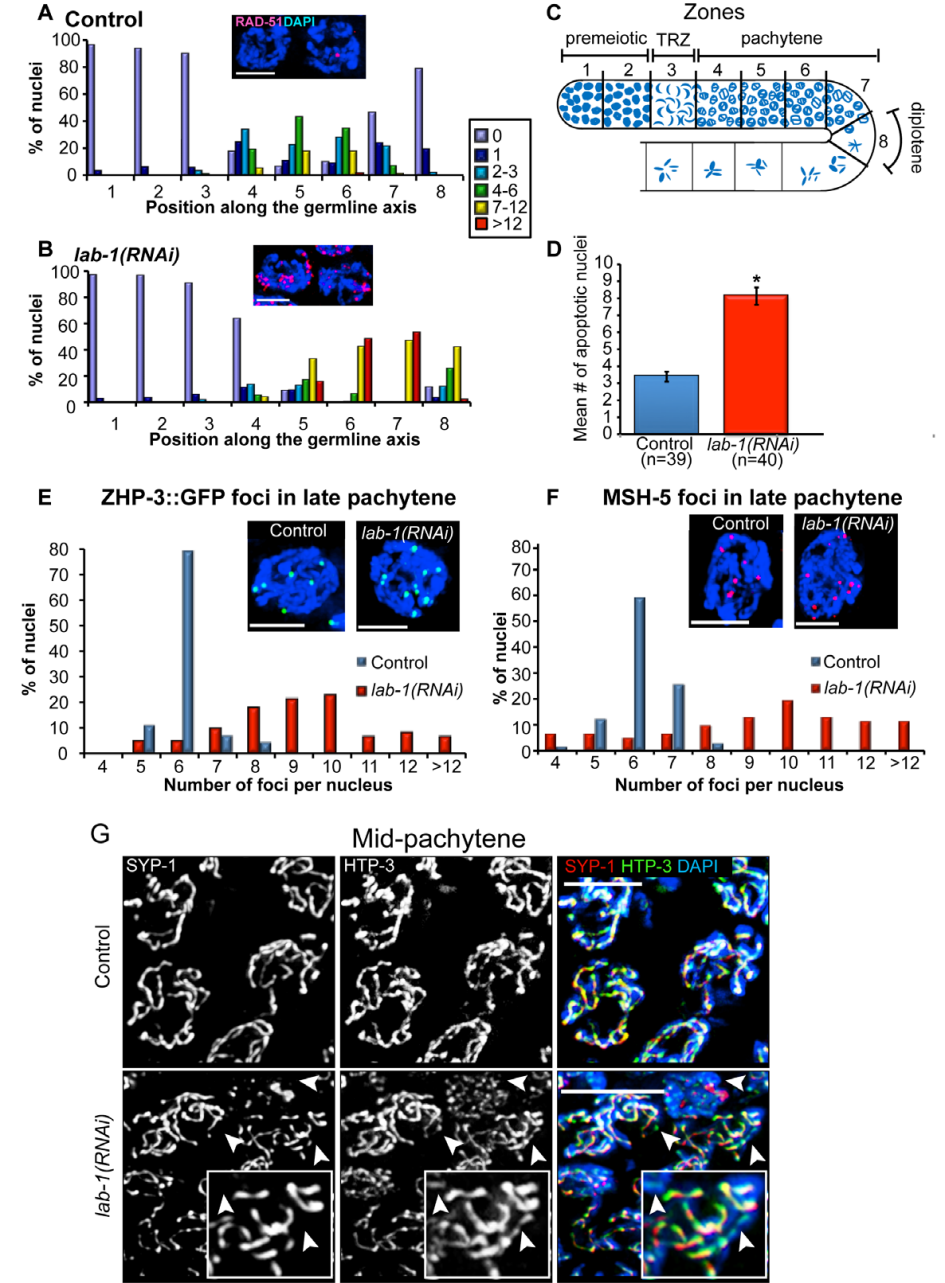
LAB-1 is required for double-strand break repair progression, crossover control, and complete synapsis. (A–B) Histograms depict the quantification of RAD-51 foci in (A) control and (B) *lab-1(RNAi)* germlines. The number of RAD-51 foci per nucleus is categorized according to the color code shown on the right. The percent of nuclei observed for each category (*y*-axis) is depicted for each zone along the germline axis (*x*-axis). Insets represent examples of late pachytene nuclei co-stained with DAPI (blue) and RAD-51 (red). Bars, 3 µM. (C) Germline diagram indicates the eight zones throughout which RAD-51 foci were scored for all nuclei. (D) Quantification of germline apoptosis. Error bars represent standard deviation of the mean. Asterisk indicates statistically significant increase in the number of apoptotic nuclei (*p*<0.0001, by the two-tailed Mann-Whitney test, 95% C.I.). *n*, number of gonad arms scored. (E–F) Histograms depict the quantification of (E) ZHP-3::GFP and (F) MSH-5 foci in late pachytene nuclei of control and *lab-1(RNAi)* germlines. Between 63 and 75 nuclei were scored from 5 to 7 gonads for each genotype. Insets depict representative nuclei (DAPI, blue; ZHP-3::GFP, green; and MSH-5, red). Bars, 3 µM. (G) Mid-pachytene nuclei in control and *lab-1(RNAi)* gonads co-stained with SYP-1 (red), HTP-3 (green), and DAPI (blue). Arrowheads indicate HTP-3-stained tracks that lack SYP-1 signal. Bars, 4 µM.

To examine whether *lab-1* depletion affects the progression of meiotic DNA double-strand break repair (DSBR), we utilized an antibody against RAD-51, a protein involved in strand invasion/exchange during DSBR [38]. In control gonads, the levels of RAD-51 foci peaked in early/mid-pachytene (zone 5), and progressively decreased in later stages (Figure 2A and 2C). In contrast, levels of RAD-51 foci were elevated throughout mid to late pachytene (zones 5–7) and persisted in 88% (*n* = 95) of early diplotene (zone 8) nuclei in *lab-1(RNAi)* gonads compared to only 21% (*n* = 97) of nuclei in control gonads (Figure 2B and Figure S2). The elevated levels of RAD-51 foci observed in *lab-1(RNAi)* gonads depend on the formation of meiotic DSBs by SPO-11 and are therefore indicative of a meiosis-specific DSBR defect (Figure S3). These results can be explained by either a delay in meiotic DSBR or an increase in the levels of DSB formation upon *lab-1* depletion. Consistent with the interpretation that nuclei in *lab-1(RNAi)* germlines contain unrepaired recombination intermediates, we observed a CEP-1/p53-dependent 2- to 3-fold increase in germ cell apoptosis in *lab-1(RNAi)* gonads compared to control, suggesting the activation of a late pachytene DNA damage checkpoint (*p*<0.0001 by the two-tailed Mann-Whitney test, 95% C.I.; Figure 2D and Figure S4). These results show that depletion of *lab-1* perturbs normal meiotic DSBR.

### Lack of LAB-1 Alters the Number of ZHP-3- and MSH-5-Marked Crossover Recombination Sites

Meiotic crossovers are tightly regulated such that at least one crossover always forms between homologs while additional crossovers nearby are discouraged [39]. Due to the decreased levels of homologous pairing observed, we hypothesized that following *lab-1* depletion, crossover levels would also be reduced. To highlight crossover precursor sites, we used a ZHP-3::GFP transgene [40],[41]. In *C. elegans*, six ZHP-3::GFP foci are observed in >78% of late pachytene nuclei, correlating with the expected one crossover event per bivalent (Figure 2E). Surprisingly, we observed a mean of 9.2 foci per nucleus in *lab-1(RNAi)* pachytene nuclei (*n* = 63, *p*<0.0001 by the two-tailed Mann-Whitney test, 95% C.I), and 4/63 had >12 foci (Figure 2E). To verify that these foci represent recombination events, we immunostained both control and *lab-1(RNAi)* gonads with an antibody that recognizes MSH-5, a conserved meiosis-specific protein required for crossover formation [42]–[45]. We found that the pattern of distribution of MSH-5 foci was almost identical to that of ZHP-3 (Figure 2F). These results suggest that *lab-1* depletion may disrupt crossover control and lead to more crossover events. Utilizing a similar cytological approach to that in Rosu et al. [46] we did not observe any bivalents with two or more chiasmata in diakinesis oocytes from *lab-1*-depleted gonads (*n* = 0/88; Figure S5). This outcome, coupled with the significant reduction in homologous pairing observed in these gonads, suggests that some of these recombination events may represent crossovers between sister chromatids as opposed to between homologs.

### Formation of the Central Region of the Synaptonemal Complex Is LAB-1-Dependent

The SC plays an essential role in promoting the maturation of DSBs into crossovers [31],[33],[34]. Therefore, we examined whether impaired chromosome synapsis might account for the altered DSBR progression observed in *lab-1(RNAi)* gonads. Specifically, we examined whether LAB-1 is required for the localization of SYP-1, a central region component of the SC. In wild type pachytene nuclei, SYP-1 is localized throughout the full length of thick DAPI-stained tracks representing paired and aligned homologous chromosomes (Figure 2G) [47]. In the *lab-1*-depleted gonads, SYP-1 signal was observed associating with chromosomes with wild type kinetics upon entry into meiosis. However, SYP-1 was not detected along the full length of DAPI-stained chromosomes in many nuclei at pachytene, as exemplified by co-staining with HTP-3, an axial element component [48] that is observed continuously throughout chromosome axes (Figure 2G). In contrast, LAB-1 is still observed localizing throughout chromosomes in *syp-1* mutants (Figure S6B). This suggests that while assembly of lateral element components of the SC is apparently LAB-1-independent at this level of cytological observation, assembly of the central region components of the SC requires LAB-1 function.

### LAB-1 Localization Depends on the Cohesin Complex

**Figure 3:**
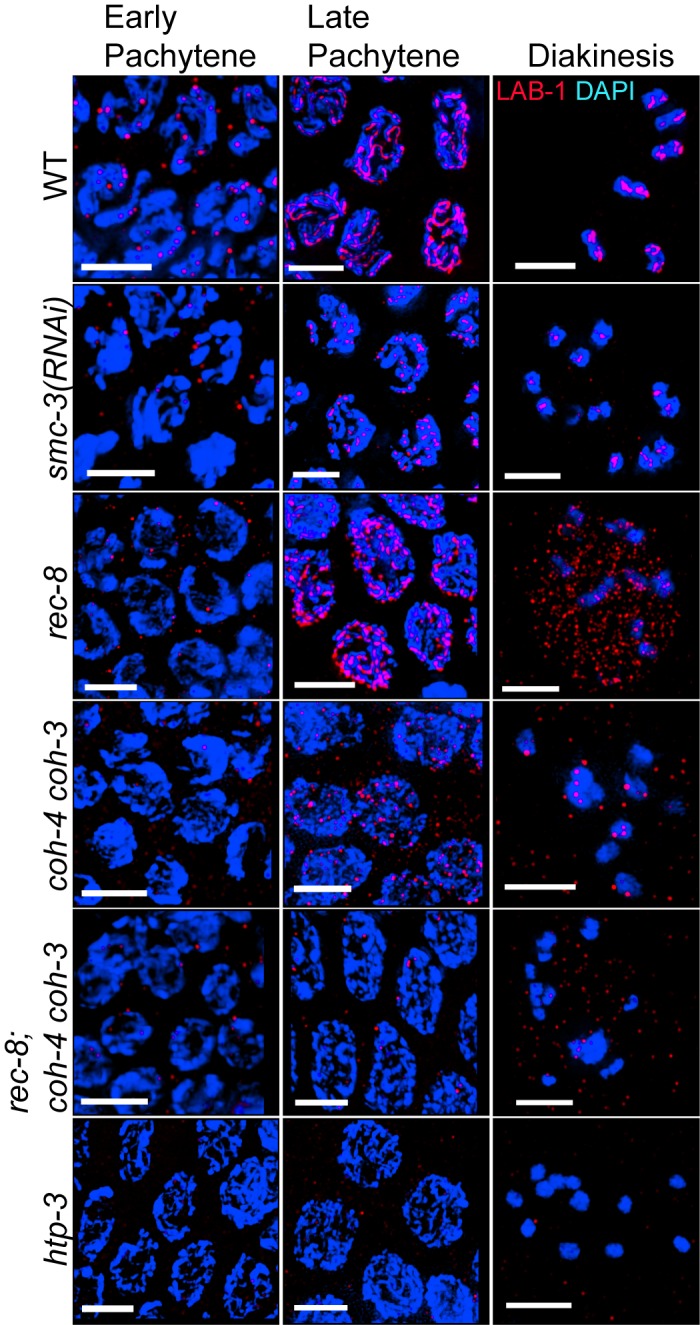
LAB-1 localization is dependent on the cohesin complex. LAB-1 (red) and DAPI (blue) staining of early pachytene, late pachytene, and −1 oocytes at diakinesis in the germlines of the indicated genotypes. Bars, 4 µM.

FISH analysis and the number of DAPI-stained bodies (>12) in diakinesis oocytes suggested that the impaired DSBR progression and chromosome synapsis observed in *lab-1(RNAi)* gonads may be due to an earlier role of LAB-1 in sister chromatid cohesion. To determine whether LAB-1 executes this role through interactions with the cohesin complex, we first examined if LAB-1 localization depends on cohesin. Depletion of the cohesin member *smc-3* resulted in an overall decrease in LAB-1 signal throughout prophase I nuclei (Figure 3). In late pachytene nuclei, only short tracks of LAB-1 were observed on the chromosomes, and LAB-1 was detected on univalents at diakinesis (Figure 3). A similar pattern was observed following depletion of the cohesin complex subunit *scc-3* (Figure S7).

We also examined whether meiosis-specific cohesin subunits were required for LAB-1 localization. In addition to REC-8, two other kleisins, COH-3 and COH-4, mediate meiotic sister chromatid cohesion in *C. elegans* [26],[49]. LAB-1 chromosomal localization was delayed in *rec-8(ok978)*, but improved as nuclei proceeded through pachytene, and by late pachytene a mixture of both long and discontinuous tracks of LAB-1 were present throughout chromosomes (Figure 3). Interestingly, LAB-1 signals were no longer associated with chromosomes in 40% (*n* = 15/37) of −1 oocytes at diakinesis, and instead were distributed diffusely throughout the nuclei of *rec-8* mutants (Figure 3). This is reminiscent of the early loss of chromosome-associated REC-8 signal observed in the *lab-1* hypomorphs on metaphase I, suggesting some degree of interdependence between these proteins [30].

In *coh-4(tm1857) coh-3(gk112)* double mutants, LAB-1 associated with the chromosomes at early pachytene, but failed to form tracks (Figure 3). When all three meiotic kleisins were mutated, LAB-1 localization was further impaired as only very few and faint LAB-1 foci were detected on either pachytene or diakinesis chromosomes (Figure 3). This effect on LAB-1 localization was specific to members of the cohesin family, as we found no change in LAB-1 localization in mutants for either *smc-5*, which is a structural maintenance of chromosomes family member, but not part of the cohesin complex, or *hcp-6*, a gene encoding a member of the condensin II complex, which is structurally similar to cohesin (unpublished data) [50]. These results suggest that LAB-1 recruitment to the chromosomes depends on the cohesin complex, and that its association with meiotic chromosomes only completely fails when all three kleisins are absent.

### LAB-1 Forms a Complex with Axial Element Proteins

To gain further insight into the function and regulation of LAB-1, we set out to identify the proteins interacting with LAB-1. Utilizing a transgenic line expressing GFP-LAB-1 and GFP antibodies for mass spectrometry analysis, we identified 19 proteins that were specifically co-immunoprecipitated from whole worm lysates with GFP-LAB-1, but not unrelated controls (Table S1). Prevalent among these were the axial element proteins HIM-3 (56.4%), HTP-1 (43.2% coverage), HTP-2 (38.9% coverage), and HTP-3 (15.8% coverage). All four proteins carry a HORMA domain that is also present in proteins involved in DSBR, synapsis, and mitotic spindle checkpoints from yeast to mammals [31],[51]–[53]. Importantly, HTP-1 is the only other known long arm-specific protein in *C. elegans* and HTP-3 was shown to be critical for meiotic sister chromatid cohesion, as multiple cohesin members fail to load onto chromosomes in *htp-3(y428)* mutants [26],[48],[54].

To assess the functional relevance of this set of interactions we examined the interdependency between the localization of LAB-1 and the HTP proteins. The localization of HTP-1, HTP-2, and HTP-3 in early prophase I was not altered in *lab-1(RNAi)* gonads compared to control (Figure 2G, Figure S6A, and Figure S6C). A reciprocal analysis revealed that LAB-1 localization is indistinguishable from wild type in *htp-1* gonads (Figure S6B). In contrast, LAB-1 signals were not observed associated with chromosomes in *htp-3* mutant germlines at any meiotic stage (Figure 3 and Figure S6D). Therefore, our analysis suggests that LAB-1 forms a complex with the HORMA domain proteins, and that HTP-3 is required for LAB-1 localization either directly or through its role in cohesin loading.

### LAB-1 and REC-8 Cooperate to Load SMC-3 and Promote SC Formation

**Figure 4:**
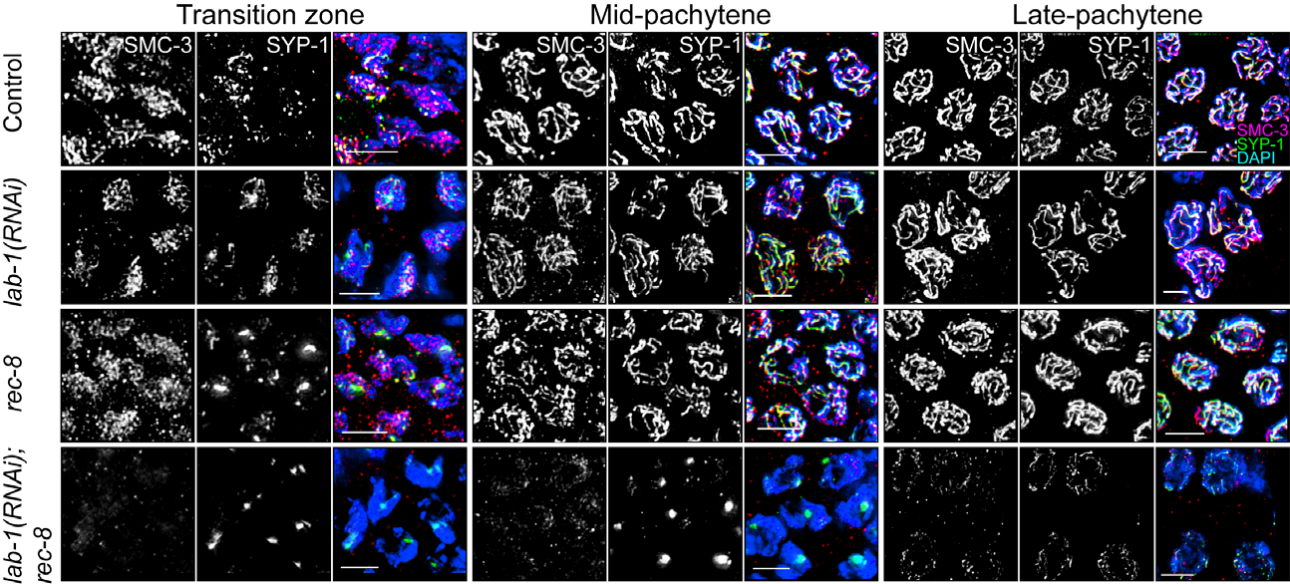
Both LAB-1 and REC-8 are required for SMC-3 and SYP-1 localization onto chromosomes. Transition zone, mid-pachytene, and late pachytene nuclei in the germlines of the indicated genotypes co-stained with SMC-3 (red), SYP-1 (green), and DAPI (blue). Bars, 4 µM.

Our findings that *lab-1* depletion results in reduced sister chromatid cohesion, and that the localization of both LAB-1 and REC-8 are partially co-dependent in late meiosis I, raise the question of whether LAB-1 could be involved in cohesin complex localization during early prophase I. Immunolocalization of either SMC-3 or REC-8 showed no differences between wild type and *lab-1*-depleted gonads (Figure 4, Figure S8, and [55]). Moreover, SMC-3 localization was indistinguishable from wild type in *rec-8* mutants (Figure 4 and [26]). Therefore, we reasoned that as both REC-8 and LAB-1 are required for normal sister chromatid cohesion, yet seem to be dispensable for SMC-3 localization, they might work in parallel. Indeed, in *lab-1(RNAi); rec-8* worms, SMC-3 loading was significantly impaired and, as expected due to abrogation of cohesin loading, the SC central region protein SYP-1 was restricted to mostly a single large aggregate per nucleus in mid-pachytene and few long tracks in late pachytene nuclei (Figure 4). Therefore, REC-8 and LAB-1 work in parallel to enable the loading of SMC-3 and facilitate SC formation.

### LAB-1 Cooperates with REC-8, COH-4, and COH-3 to Ensure Sister Chromatid Cohesion

**Figure 5:**
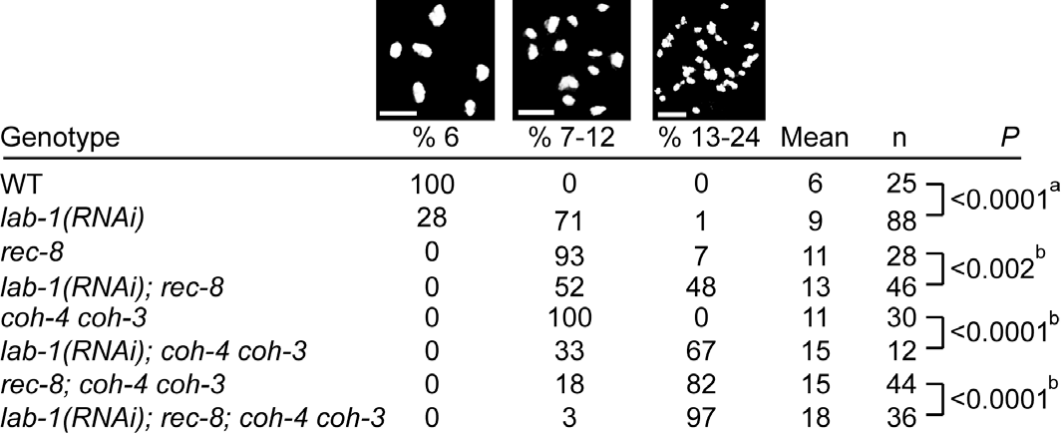
*lab-1* depletion weakens sister chromatid cohesion. Number of DAPI-stained bodies in the −1 oocyte at diakinesis in the indicated genotypes. The *p* values were calculated by (a) the Wilcoxon signed-rank test and by (b) the two-tailed Mann-Whitney test, 95% C.I. Bars, 4 µM.

If LAB-1 and REC-8 cooperate in SMC-3 loading during meiosis, then lack of both should increase the premature loss of sister chromatid cohesion detected at diakinesis. Indeed, the number of diakinesis oocytes carrying 13–24 DAPI stained bodies is significantly increased in *lab-1(RNAi); rec-8* gonads compared with either *lab-1(RNAi)* or *rec-8* (48%, 1%, and 7%, *n* = 46, 88, and 28, respectively). Nevertheless, many chromatids were still held together in *lab-1(RNAi); rec-8* as demonstrated by the 52% of oocytes that had 7–12 DAPI stained bodies (average 13.1±2.5; Figure 5). This could be explained by either residual LAB-1 that was not depleted or by other factors that contribute to sister chromatid cohesion independently. To examine whether the other two meiotic kleisins contribute to sister chromatid cohesion in parallel with *lab-1*, we looked at the effects of *lab-1* depletion on *coh-4 coh-3* double mutants. 7–12 DAPI stained bodies are observed in the diakinesis oocytes of *coh-4 coh-3* double mutants (Figure 5 and [26]), suggesting that sister chromatids are still held together. However, when we depleted *lab-1* in these worms, the average number of DAPI-stained bodies increased from 11±1 to 15±3 (Figure 5, *n* = 30 and 12, respectively, *p*<0.0001 by the two-tailed Mann-Whitney test, 95% C.I). These results suggest that LAB-1 affects both REC-8 and COH-3/COH-4 cohesin complexes. Moreover, the role of LAB-1 in SCC may be greater than suggested by these results, since this analysis relied on RNAi depletion and therefore residual LAB-1 activity cannot be ruled out.

Two models for *lab-1* contribution to sister chromatid cohesion can be envisioned from these results. In one, *lab-1* ensures sister chromatid cohesion solely through *rec-8*, *coh-4*, and *coh-3*. Alternatively, *lab-1* can contribute to sister chromatid cohesion even in their absence. To distinguish between these two possibilities, we examined the effect of *lab-1* depletion when all three meiotic kleisins are mutated. Similar to previous observations [26], we found that most of the oocytes at diakinesis in the *rec-8;coh-4 coh-3* worms had 13–24 DAPI stained bodies (Figure 5). However, the average number of bodies was only 15±3, indicating that sister chromatid cohesion was not completely lost. When *lab-1* was depleted in these worms, the number of DAPI-stained bodies increased to 18±3, and 97% had 13–24 bodies (Figure 5, *n* = 44 and 36 for control and *lab-1(RNAi)*, respectively, *p*<0.0001 by the two-tailed Mann-Whitney test, 95% C.I.). This result suggests that *lab-1* acts to promote sister chromatid cohesion in parallel with *rec-8*, *coh-4*, and *coh-3*.

### LAB-1 Directly Interacts with the PP1 Homologs

**Figure 6:**
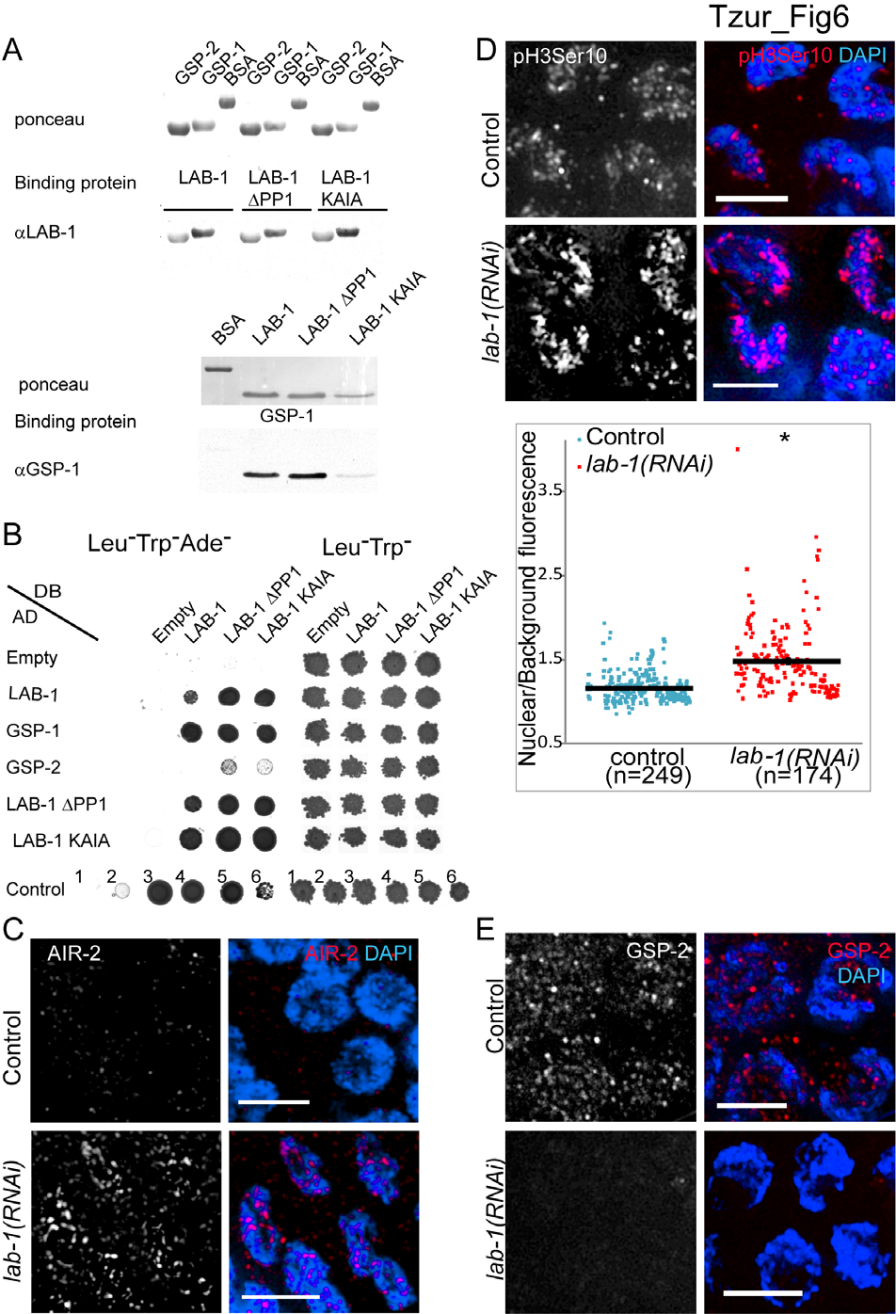
LAB-1 binds GSP-1 and GSP-2 and is required for correct AIR-2 and GSP-2 localization on early meiotic nuclei. (A) Far-western analysis of in vitro binding of purified recombinant LAB-1, LAB-1 PP1 putative motif mutants, GSP-1, and GSP-2. Purified bacterially expressed proteins were transferred to membranes, incubated with the indicated binding protein, and probed with appropriate antibodies. BSA was used as control. (B) The yeast two-hybrid system was used to test the protein interactions between LAB-1, LAB-1 PP1 putative motif mutants, GSP-1, and GSP-2. Proteins were fused to either the DNA binding domain (DB) or the activation domain (AD) of GAL4. Interactions were scored by growth on SC-Leu-Trp-Ade plates. Growth on SC-Leu-Trp was used as a control. Negative (No. 1) and positive (No. 2–6) controls are shown. Positive interactions are shaded in gray. (C) Transition zone nuclei in control and *lab-1(RNAi)* gonads co-stained with AIR-2 (red) and DAPI (blue). (D) Transition zone nuclei in control and *lab-1(RNAi)* gonads co-stained with H3S10ph (red) and DAPI (blue). A quantification plot is presented below where each dot represents the ratio between the level of fluorescence detected for an individual nucleus and that of the adjacent background. Error bars represent standard deviation of the mean. Asterisk indicates statistically significant increase in fluorescence (*p*<0.0001, by the two-tailed Mann-Whitney test, 95% C.I.). (E) Transition zone nuclei in control and *lab-1(RNAi)* gonads co-stained with GSP-2 (red) and DAPI (blue). Bars, 4 µM.

We have previously hypothesized that LAB-1 targets the PP1 homologs GSP-1 and GSP-2 to the long arms of the bivalents, thereby antagonizing AIR-2 localization to that region [30]. To test whether LAB-1 can directly interact with either PP1 homolog we utilized the far-western assay [56]. Bacterially expressed and purified GSP-1 and GSP-2 proteins transferred to nitrocellulose membranes bound purified LAB-1 (Figure 6A). Reciprocally, LAB-1 transferred to membranes bound GSP-1 (Figure 6A). These results suggest that LAB-1 binds to the PP1 homologs in vitro.

A highly degenerate motif [(R/K) (V/I) X (F/W)] has been previously associated with the binding, localization, and function of PP1 phosphatases [57]–[59]. To test if the putative PP1 binding motif present in LAB-1 [30] is required for the in vitro interaction detected between LAB-1 and the PP1 homologs, we used purified LAB-1 protein either lacking the motif (ΔPP1) or carrying two alanine substitutions in this motif (KAIA). GSP-1 and GSP-2 proteins transferred to membranes were still able to bind both mutant LAB-1 proteins, and reciprocally, membrane-bound mutant LAB-1 proteins could bind GSP-1 (Figure 6A). Thus, the putative PP1 binding motif is probably not necessary for LAB-1 binding to GSP-1 and GSP-2.

To verify the interaction between LAB-1 and the PP1 homologs we utilized the yeast two-hybrid system. Full-length GSP-1 fused to the activation domain of GAL4 interacted with LAB-1 as well as with the LAB-1 PP1 mutants fused to the DNA binding domain of GAL4 (Figure 6B). Interestingly, GSP-2 could only weakly interact with the LAB-1 PP1 mutants but not with wild type LAB-1 (Figure 6B). It is possible that LAB-1 binding to GSP-2 is masked in yeast cells by the host's endogenous PP1 homologs, and only when these unrelated interactions are removed, LAB-1 and GSP-2 interaction can be detected. Taken together, these results support a direct interaction between LAB-1 and the PP1 homologs.

### LAB-1 Is Required to Target GSP-2 to Early Meiotic Nuclei and Restrict AIR-2

The mislocalization of AIR-2 to the long arms of the bivalents during diakinesis in *lab-1* hypomorph mutants [30] prompted us to test whether depletion of *lab-1* by RNAi results in changes in AIR-2 localization during early meiotic stages as well. Indeed, unlike in most control gonads (*n* = 45/53), in which AIR-2 signal was not observed in transition zone nuclei, clear AIR-2 patches were observed in most transition zone nuclei upon *lab-1* depletion (*n* = 16/30) (Figure 6C, *p*<0.0005, by the two-sided Fisher's Exact Test, 95% C.I.). Moreover, consistent with AIR-2 localization in early prophase I, increased histone H3 phosphorylation, a well-characterized chromosomal target for Aurora B was also observed at that stage (Figure 6D, mean relative fluorescence = 1.2, *n* = 284, and 1.5, *n* = 175, for control and *lab-1(RNAi)*, respectively; *p*<0.0001 by the two-tailed Mann-Whitney test, 95% C.I.) [24],[60]. Since LAB-1 binds the PP1 homologs and was suggested to restrict AIR-2 through GSP-2 in metaphase I [30], we tested if depletion of *lab-1* also changes GSP-2 localization in early meiotic nuclei. In control gonads, GSP-2 is found in foci throughout transition zone nuclei and the syncytial gonad (*n* = 12/12) (Figure 6E and Figure S9). However, in most *lab-1* depleted gonads (*n* = 7/12), the level of nuclei-associated GSP-2 foci was reduced (Figure 6E and Figure S10, *p*<0.05, by the two-sided Fisher's Exact Test, 95% C.I.). These results suggest that LAB-1 directly targets GSP-2 to the chromatin during meiotic onset, thus restricting aberrant AIR-2 accumulation at that stage and promoting normal establishment and maintenance of sister chromatid cohesion.

## Discussion

### LAB-1 Is Required for Sister Chromatid Cohesion

The dynamic nature of chromosome interactions during the cell cycle requires the ability to establish, mobilize, and remove SCC. Since the discovery of the core components of the cohesin complex, a growing number of proteins have been found to take part in all aspects of cohesin function: proteins involved in loading cohesin, maintaining its binding, and removing it from chromosomes. The importance of SCC is highlighted in meiosis, due to the intricacy and complexity of this process. During prophase I, homologous chromosomes pair, synapse, undergo programmed meiotic DSBs and recombine [32]. Thus, while a small reduction in chromatid cohesion may still provide for normal mitotic segregation, it would be unable to support the structural requirements for the meiotic processes to proceed. For example, in the yeast *smc3-42* temperature-sensitive mutant, both SC and crossover formation are perturbed and cells fail to undergo any meiotic division, even in the mitotic permissive temperature [61]. In *C. elegans*, lack or depletion of either cohesin members or their interactors have also been shown to alter pairing, synapsis, DSBR, and accurate chromosome segregation [26],[55],[62]–[64]. Another level of cohesin control must be employed in meiosis due to the requirement for protection of cohesin at specific chromosomal regions, namely at centromeres in the case of monocentric organisms and along the long arms of the bivalents in the case of the holocentric *C. elegans* chromosomes. Along the long arms, cohesin must be preserved during the first meiotic division, while in all other parts of the chromosomes it must be removed [6],[29],[65]. Without this protection, faithful chromatid segregation cannot take place during the second meiotic division. It is therefore not surprising that various different components are involved in regulating SCC. However, how these proteins modulate the way SCC is either enforced or relieved, and how these two processes are coordinated, is not completely understood.

Most of the factors implicated in SCC depend on the cohesin complex, yet some reports have also suggested cohesin-independent pathways [66]–[68]. Although LAB-1 can maintain some degree of SCC even in the absence of the meiotic kleisins, most of our data support a cohesin-dependent mechanism. Here we show that LAB-1 and different cohesin members are partially interdependent in their localization throughout prophase I and at metaphase I. The cooperation between LAB-1 and cohesin to ensure SCC can be observed cytologically through the number of DAPI-stained bodies at diakinesis when meiotic kleisins are mutated. Mutations in either *rec*-8 or *coh-4 coh*-3 do not result in precocious sister separation in most nuclei, yet depletion of *lab-1* in those mutants significantly increases the frequency of unbound sister chromatids at diakinesis. These results are consistent with LAB-1 acting to maintain cohesion in cooperation with the cohesin complex. Yet it is possible that LAB-1 can promote SCC in a pathway that does not require the meiotic kleisins, since we observed a significant increase in sister chromatid separation when we depleted *lab-1* in the *rec-8*, *coh-3*, and *coh-4* triple mutant. The factors taking part in this pathway remain to be uncovered. Interestingly, our preliminary analysis did not reveal increased loss of sister chromatid cohesion following *lab-1* depletion in either *scc-1* or *smc-3* depleted backgrounds (Y.B.T. and M.P.C. unpublished results). However, the lack of fully separated chromatids in the *rec-8; coh-4 coh-3* triple mutant could be due to mechanisms involving yet other kleisins and/or the formation of tangles between sister chromatids.

Yan and colleagues have recently reported the finding of sisters on the loose (SOLO), a protein that together with stromalin on meiosis (snm) is required for centromere cohesion and SMC1 localization during Drosophila male meiosis [69]. We suggest that LAB-1 plays a similar role, in that it is required to both appropriately load and maintain the cohesin complexes on specific chromosomal subdomains during pre-meiotic S phase and prophase I, respectively, in *C. elegans*, thereby enabling the meiotic program. In this context (Figure S11), LAB-1 acts to properly load and maintain the association of cohesin complexes along the chromosome axes. Therefore, the meiotic defects observed in *lab-1(RNAi)* gonads are diagnostic of problems in SCC. First, lack of LAB-1 perturbs homolog paring. In this state, interhomolog repair is impaired, DSBR progression is altered and many nuclei undergo apoptosis. Second, nuclei that are not eliminated by apoptosis contain univalents as well as single chromatids at diakinesis. Finally, in the bivalents where SCC is not lost in early prophase I, reduced LAB-1 on the long arm is ultimately insufficient to prevent unchecked AIR-2 loading on all chromosome axes [30]. Upon metaphase I entry, REC-8 removal occurs at both short and long arms and accurate homolog segregation fails (Figure S11).

How does LAB-1 affect SCC? Our finding that LAB-1 and HTP-3 are present in the same complex raises the possibility that LAB-1 acts to maintain SCC via HTP-3-mediated control of cohesin loading [26]. Alternatively, HTP-3 may target LAB-1 either directly or indirectly to chromosome axes, and LAB-1 in turn recruits other factors that maintain SCC. The presence of other axial element proteins in LAB-1 immunoprecipitates as well as our finding that LAB-1 directly interacts with the PP1 homologs lead us to favor the latter.

We found that in the yeast two-hybrid system GSP-2 could only weakly interact with the LAB-1 protein carrying mutations in the putative PP1 binding motif, leading us to hypothesize that GSP-2 is a weaker binding partner of LAB-1 than GSP-1. Weak or transient binding is probably the reason why we did not detect GSP-1 and GSP-2 in our LAB-1 IPs, which were done using stringent conditions. Nevertheless, *lab-1* depletion affected the localization of GSP-2, suggesting that LAB-1 indeed targets GSP-2 to early meiotic nuclei.

### Early Versus Late Roles for LAB-1 During Meiosis I

**Figure 7:**
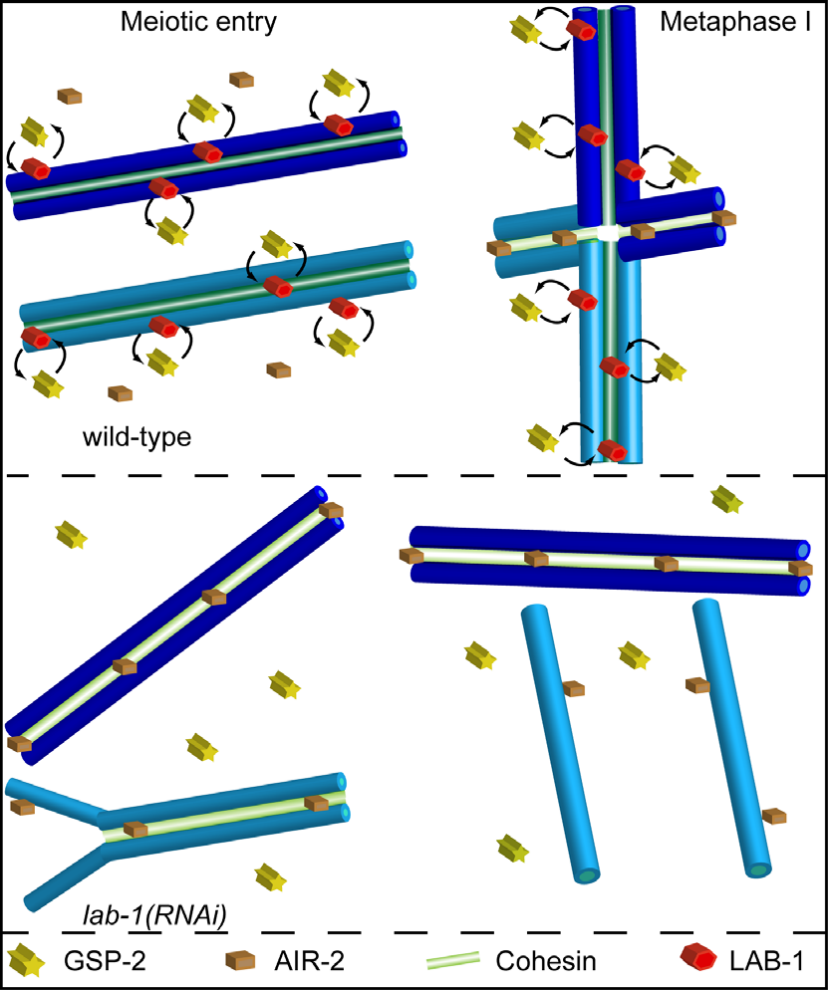
LAB-1 utilizes a similar mechanism to protect SCC during two different meiotic stages. In wild type, LAB-1 starts associating with the chromosomes at the entry into meiosis and transiently targets GSP-2 to the chromosomes. This targeting antagonizes AIR-2 and maintains SCC. During diakinesis, LAB-1 localizes to the long arms, where it specifically antagonizes AIR-2 and protects REC-8 from premature removal. When *lab-1* is depleted, AIR-2 associates with the chromosomes as early as transition zone, and SCC is perturbed. The weakened SCC prevents successful binding of homologs and many reach diakinesis as either univalents or detached chromatids.

LAB-1 localization in the germline is highly dynamic. We propose that at the onset of meiosis, the chromosomal association of LAB-1 opposes cohesin-removing proteins whose levels gradually increase during pachytene. Indeed, AIR-2 signal is only first observed in late pachytene in wild-type *C. elegans* [23], whereas it accumulates in transition zone nuclei following *lab-1* depletion (this work). Given that AIR-2 promotes cohesin removal during mitotic prophase [24],[70]–[74], we suggest that a role for LAB-1 is to restrict AIR-2's ability to remove cohesin during early meiosis. According to this model, the system that protects cohesin during metaphase I, and involves LAB-1 antagonizing AIR-2 probably via the PP1s along the long arms of the bivalents, also operates throughout the early stages of meiosis to maintain SCC (Figure 7). This may be achieved by targeting the PP1s to chromosome axes and restricting AIR-2. In late prophase I, LAB-1 is lost from part of the chromosomes, permitting the removal of cohesin at these subdomains.

In this article we show that LAB-1 plays an important role in protecting sister chromatid cohesion by localizing a phosphatase (PP1 by LAB-1 instead of PP2A by Shugoshin) and antagonizing Aurora B phosphorylation activity. Thus, our studies have revealed key conserved principles that guide proper regulation of meiotic sister chromatid cohesion. The use of PP1 to maintain and protect cohesin, while possibly the result of a necessary co-evolution with the holocentric nature of *C. elegans* chromosomes, may also be required for monocentric organisms. Similar to LAB-1, Sgo2 was found to maintain cohesion in mouse bivalents during late prophase I [75]. This raises the possibility that Shugoshin proteins in other metazoans may also play a role earlier in meiosis in establishing and/or maintaining sister chromatid cohesion. In support of this possibility, it was recently shown that PP2A has early meiotic roles, in addition to its role of protecting centromeric cohesin during metaphase I [76]. Therefore, the use of mouse meiotic conditional alleles may aid in assessing the effect of these proteins in early prophase I.

In conclusion, we have shown that LAB-1 has an earlier role in regulating the establishment and maintenance of SCC. Thus, LAB-1 emerges as a central protein in the regulation of SCC, exerting this role from the start of prophase I through homolog segregation at the metaphase I to anaphase I transition.

## Materials and Methods

### Strains and Alleles

The N2 Bristol strain was used as the wild-type background. *C. elegans* strains were cultured at 20 °C under standard conditions as described in [77]. The following mutations were used: LGI: *htp-3(tm3655)*, *cep-1(lg12501)*, *lab-1(tm1791)*, LGIV: *htp-1(gk174)*, *rec-8(ok978)*, *spo-11(ok79)*, LGV: *coh-4(tm1857)*, *coh-3(gk112)*, and *syp-1(me17)* ([26],[30],[35],[47],[78]–[80]). The GFP::LAB-1::HA line has been previously described in [30].

### RNAi

Feeding RNAi experiments were performed at either 20 °C (for *smc*-3 and *scc-3*) or 25 °C (for *lab-1*) as described in [30],[63],[81]. Control RNAi was performed by feeding HT115 bacteria carrying the empty pL4440 vector. A feeding vector from the ORFeome RNAi collection [82] was used for *smc-3* RNAi experiments. Successful depletions were verified at every single experiment by immunostaining with either LAB-1 or SMC-3 antibodies for *lab-1(RNAi)* and *smc-3(RNAi)*, respectively, and by RT-PCR for *scc-3(RNAi)*.

### RT-PCR

cDNA was produced from single-worm RNA extracts using the Thermoscript RT-PCR system (Invitrogen). The effectiveness of *scc-3* RNAi was determined by assaying the expression of the *scc-3* transcript in at least four individual animals subjected to RNAi. Expression of *gpd-1* (T09F3.3) was used as a control.

### Immunostaining, DAPI Analysis, and FISH

Whole mount preparation of dissected gonads, DAPI staining, immunostaining, and analysis of germline nuclei were carried out as in [41],[83]. A rabbit polyclonal antibody against a C-terminal peptide of *C. elegans* GSP-2 (TPPRNAPAAQPKKGAKK) was generated by Sigma-Genosys. The antiserum was affinity-purified against the original peptide-antigen as described in [84]. Primary antibodies were used at the following dilutions: α-LAB-1 (1∶300; [30]), α-REC-8 (1∶50; Abcam), α-Histone H3 phospho-Ser10 (1∶300; Upstate Biotechnologies), α-HTP-3 (1∶100; [48]), α-HTP-1/2 (1∶200; [54]), α-SYP-1 (1∶200 [47]), α-RAD-51 (1∶100; [83]), α-SMC-3 (1∶500; Chemicom), α-MSH-5 (1∶100000; SDI), α-AIR-2 (1∶100 [30]), and α-GSP-2 (1∶100). The secondary antibodies used were: Cy3 α-rabbit, Cy3 α-rat, Cy3 α-mouse, FITC α-rabbit, and FITC α-guinea pig (Jackson Immunochemicals, 1∶200).

FISH was performed as in [37] utilizing a probe recognizing the left end of the X chromosome, derived from YAC Y51E2, and a probe recognizing the right end of chromosome I derived from pooled cosmids F32A7 and F14B11, prepared as in [36].

### Imaging, Microscopy, and Mass Spectrometry

Immunofluorescence images were collected at 0.2 µm increments with an IX-70 microscope (Olympus) and a cooled CCD camera (model CH350; Roper Scientific) controlled by the DeltaVision system (Applied Precision). Images were subjected to deconvolution analysis using the SoftWorx 3.0 program (Applied Precision) as in [23]. For germ cell apoptosis, worms were transferred onto a drop of M9 on 1.5% agarose pads on slides and assayed using the Leica DM5000 B microscope (100× objective). Mass spectrometry analysis was done as described in [84]. Specificity was further supported by analysis of >7 other *C. elegans* proteins precipitated in the same approach (I. Cheeseman, personal communication and [85]–[87]).

### Quantification of Immunofluorescence Signal

Control and *lab-1(RNAi)* worms were mounted on the same slides, but either the heads or tails were dissected to distinguish between genotypes. Immunostaining and imaging were performed as described above. Images were acquired from gonads still attached to carcasses. Fluorescence intensity was measured using ImageJ. Values were normalized by dividing the fluorescence intensity level detected in a rectangular area encompassing each nucleus with the intensity level detected within the same size area of the adjacent cytoplasm.

### Far-Western and Yeast Two-Hybrid Assays

For far-westerns, 0.8 µg (as determined by Micro BCA protein assay kit, Pierce Biotechnology Rockford, IL) of each protein were resolved on 12.5% SDS-PAGE and transferred to nitrocellulose membranes. Membranes were stained with Ponceau S and then washed with PBS. The membranes were then blocked for 1 h in PBSTBM (PBS containing 1% Tween 20, 5% dry milk, and 1% BSA), and incubated overnight with 2 µg/ml of the appropriate binding protein diluted in PBSTBM at 4°C. After four washes with PBSTBM, the membranes were incubated for 1 h at RT with primary antibody diluted 1∶100,000 in PBSTMB for 1 h, washed four times with PBST, and incubated for 1 h with peroxidase-conjugated secondary antibodies diluted 1∶10,000 in PBST. Incubation with an N-terminus HIM-18 antibody was used as a negative control (Figure S12).

Yeast two-hybrid assays were performed as in [41].

### Time Course Analysis for RAD-51 Foci

Quantitative analysis of RAD-51 foci was performed as in [83] except that eight instead of seven zones composing the germline were scored. The eighth additional zone included in this study consists of early diplotene nuclei. The average number of nuclei scored per zone (*n*) from six gonads each for control and *lab-1(RNAi)* were: zone 1 (*n* = 247), zone 2 (*n* = 311), zone 3 (*n* = 267), zone 4 (*n* = 204), zone 5 (*n* = 181), zone 6 (*n* = 160), zone 7 (*n* = 134), and zone 8 (*n* = 96).

### Quantitative Analysis of Germ Cell Apoptosis

Germ cell corpses were scored in adult hermaphrodites 18 h post-L4 using acridine orange as described in [43]. A minimum of 35 gonads were scored for each genotype. Statistical analysis was performed using the two-tailed Mann-Whitney test, 95% C.I.

## Supporting Information

Figure S1 *lab-1* depletion by RNAi results in complete loss of LAB-1-specific immunofluorescence signal. Late pachytene nuclei in control and *lab-1(RNAi)* gonads co-stained with LAB-1 (red) and DAPI (blue). The weak residual signals observed in *lab-1(RNAi)* are unspecific and not associated with chromatin. Bars, 4 µM.
(TIF)

Figure S2 Depletion of *lab-1* increases the mean number of RAD-51 foci during prophase I. Histograms depict the quantification of the mean number of RAD-51 foci observed per nucleus (*y*-axis) along the germline axis of both control and *lab-1(RNAi)* worms. Error bars represent standard deviation of the mean.
(TIF)

Figure S3 Levels of RAD-51 foci are elevated in a SPO-11-dependent manner in *lab-1(RNAi)* germlines. Histograms depict the quantification of RAD-51 foci in (A) control and (B) *lab-1(RNAi)* in *spo-11* germlines. The number of RAD-51 foci per nucleus is categorized according to the color code shown on the right. The percent of nuclei observed for each category (*y*-axis) are depicted for each zone along the germline axis (*x*-axis).
(TIF)

Figure S4 Increased germ cell apoptosis in *lab-1(RNAi)* worms is CEP-1/p53-dependent, indicating activation of a DNA damage checkpoint. Quantification of germline apoptosis by scoring acridine orange positive nuclei in control, *cep-1* control, *lab-1(RNAi)*, and *cep-1 lab-1(RNAi)* worms. Error bars represent standard deviation of the mean. *n*, number of gonad arms scored.
(TIF)

Figure S5 Detection of chiasmata in *lab-1*-depleted gonads. Partial projection of a *z* stack of images collected from a diakinesis nucleus in a *lab-1(RNAi)* gonad co-stained with HTP-3 (green) and DAPI (blue). Arrowheads point towards bivalents in which a single chiasma is clearly detected by the cruciform organization of the axes highlighted by HTP-3. Bar, 4 µM.
(TIF)

Figure S6 Interdependency analysis of chromosomal localization of the HTP-1/-2/-3 and LAB-1 proteins. (A) Mid-pachytene nuclei in control and *lab-1(RNAi)* gonads co-stained with HTP-1/2 (red) and DAPI (blue). (B) Late-pachytene nuclei in wild type, *htp-1*, and *syp-1* mutants co-stained with LAB-1 (red) and DAPI (blue). (C) Transition zone nuclei in control and *lab-1(RNAi)* gonads co-stained with HTP-3 (green) and DAPI (blue). (D) Transition zone nuclei in wild type, and *htp-3* mutants co-stained with LAB-1 (red) and DAPI (blue). Bars, 4 µM.
(TIF)

Figure S7 LAB-1 localization is SCC-3-dependent. High-magnification images of early pachytene and late pachytene nuclei as well as −1 oocytes at diakinesis co-stained with LAB-1 (red) and DAPI (blue) in *scc-3(RNAi)* gonads. Bars, 4 µM.
(TIF)

Figure S8 REC-8 localization in early prophase I is not altered following *lab-1* depletion. High-magnification images of transition zone, mid-pachytene, and late pachytene nuclei co-stained with REC-8 (green) and DAPI (blue) in control and *lab-1(RNAi)* germlines. Bars, 4 µM.
(TIF)

Figure S9 Specificity of GSP-2 antibodies. High-magnification images of pachytene nuclei co-stained with GSP-2 (red) and DAPI (blue) in wild-type and *gsp-2* germlines. Bars, 4 µM.
(TIF)

Figure S10 GSP-2 signal associated with transition zone nuclei is LAB-1-dependent. Transition zone nuclei in control and *lab-1(RNAi)* gonads mildly squashed as in [83], and co-stained with GSP-2 (red) and DAPI (blue). Bars, 4 µm.
(TIF)

Figure S11 Depletion of *lab-1* SCC reduction manifests as meiotic defects. We propose that LAB-1 assists in cohesin loading in the pre-meiotic region, and maintenance of the complex during early prophase I, whereas it protects REC-8 from premature removal at the long arms of the bivalents at metaphase I. When *lab-1* is depleted, cohesin is not loaded correctly, potentially creating localized regions with either a lack or reduction of cohesin. The partial dissociation of sister chromatids reduces homologous pairing and impairs the repair of DSBs via interhomolog recombination, possibly due to the lack of a stable homologous template in close proximity. This results in either apoptosis or the use of alternative modes of meiotic DSB repair, such as intersister-based repair. Upon remodeling, the lack of both SCC and interhomolog crossovers leads to the formation of both univalents and single chromatids. Lack of LAB-1 in metaphase I results in the premature removal of REC-8 from the long arms and increased errors in chromosome segregation.
(TIF)

Figure S12 LAB-1 can specifically bind GSP-1 and GSP-2 in vitro. Far-western assay for in vitro binding of purified recombinant LAB-1 and N-HIM-18 (negative control) to GSP-1 and GSP-2 transferred to membranes.
(TIF)

Table S1 LAB-1 interacting proteins. Immunoprecipitation (IP) from LAB-1::GFP whole worm extracts with an antibody against GFP was analyzed by mass spectrometry. Numbers indicate the total mass spectra collected in two samples.
(DOC)

## Abbreviations

AD: activation domain
DB: DNA binding domain
DSB: double-strand break
DSBR: double-strand break repair
FISH: fluorescence in situ hybridization
PP1: protein phosphatase 1
PP2A: protein phosphatase 2A
SC: synaptonemal complex
SCC: sister chromatid cohesion
