# Supplementary figures and images for: LAB-1 Targets PP1 and Restricts Aurora B Kinase upon Entrance into Meiosis to Promote Sister Chromatid Cohesion
Source: PLoS Biol. 2012 Aug 21;10(8):e1001378. doi: 10.1371/journal.pbio.1001378 (PMC3424243; doi:10.1371/journal.pbio.1001378)

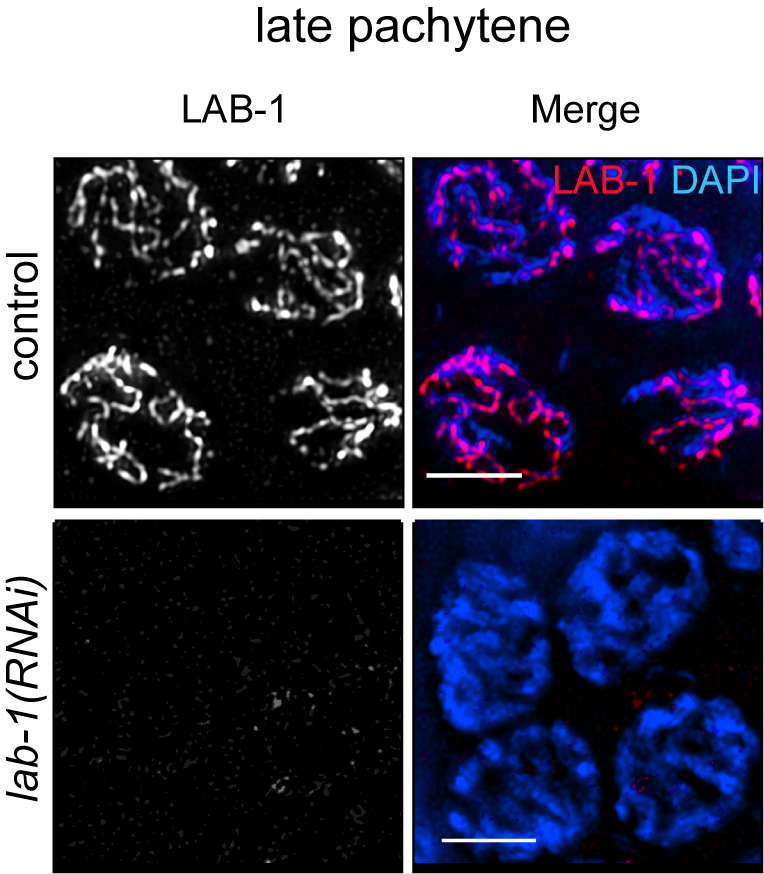

Supplement: Figure S1 — lab-1 depletion by RNAi results in complete loss of LAB-1-specific immunofluorescence signal. Late pachytene nuclei in control and lab-1(RNAi) gonads co-stained with LAB-1 (red) and DAPI (blue). The weak residual signals observed in lab-1(RNAi) are unspecific and not associated with chromatin. Bars, 4 µM. (TIF) [file pbio.1001378.s001.tif]

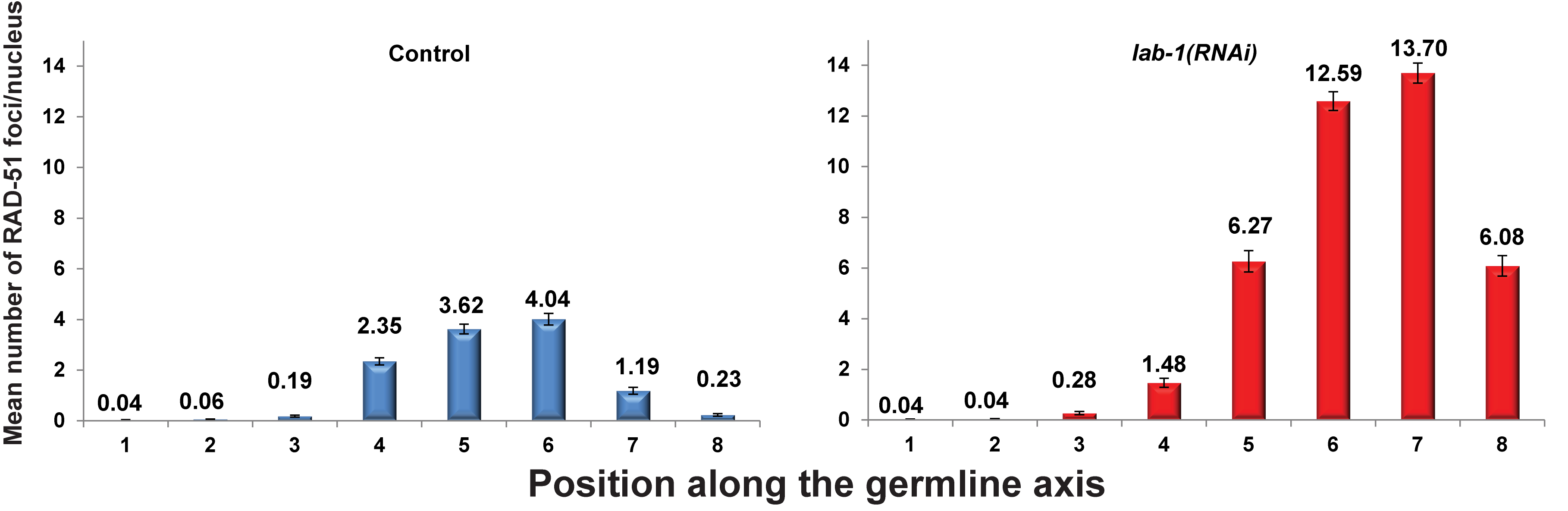

Supplement: Figure S2 — Depletion of lab-1 increases the mean number of RAD-51 foci during prophase I. Histograms depict the quantification of the mean number of RAD-51 foci observed per nucleus (y-axis) along the germline axis of both control and lab-1(RNAi) worms. Error bars represent standard deviation of the mean. (TIF) [file pbio.1001378.s002.tif]

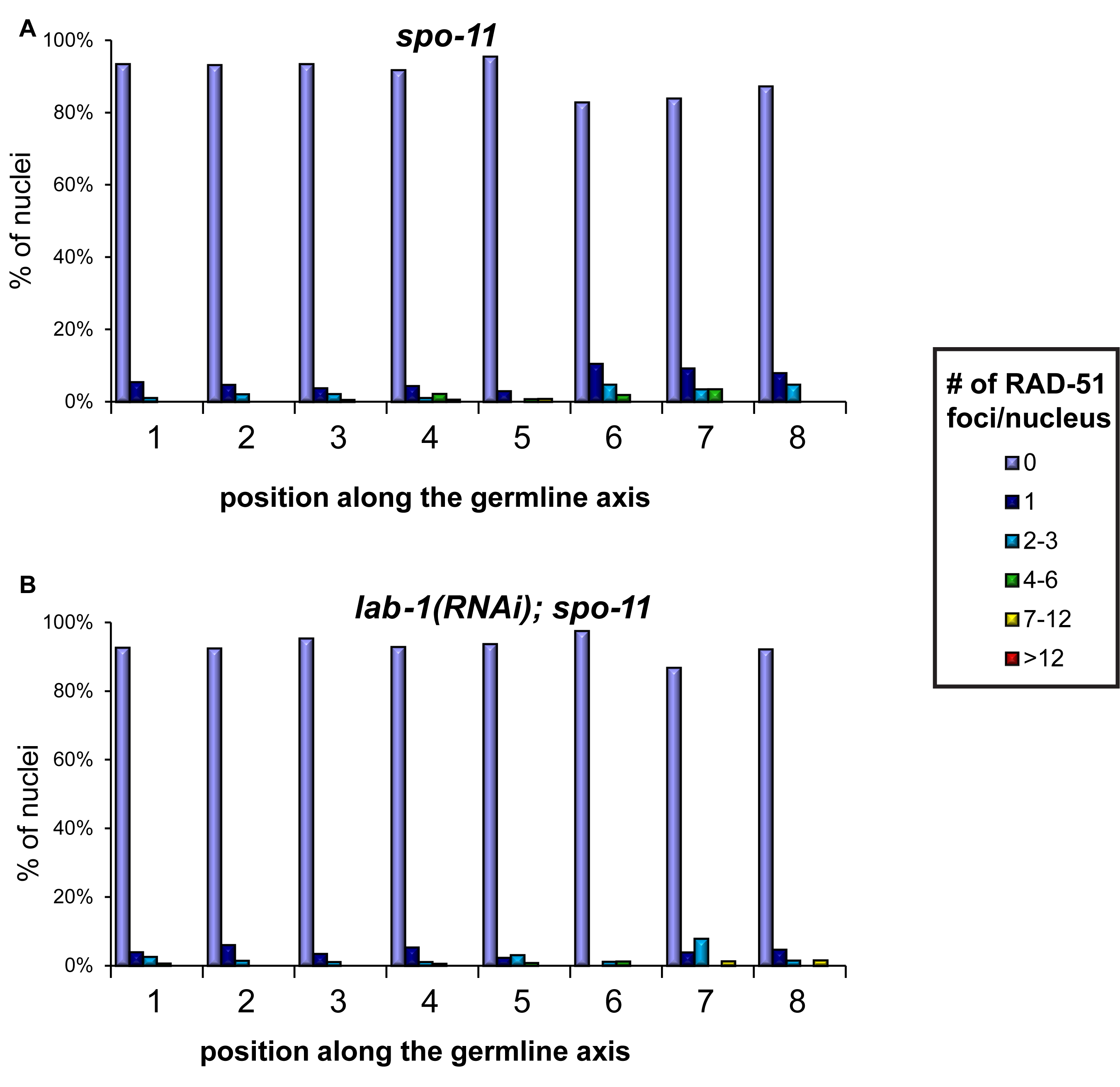

Supplement: Figure S3 — Levels of RAD-51 foci are elevated in a SPO-11-dependent manner in lab-1(RNAi) germlines. Histograms depict the quantification of RAD-51 foci in (A) control and (B) lab-1(RNAi) in spo-11 germlines. The number of RAD-51 foci per nucleus is categorized according to the color code shown on the right. The percent of nuclei observed for each category (y-axis) are depicted for each zone along the germline axis (x-axis). (TIF) [file pbio.1001378.s003.tif]

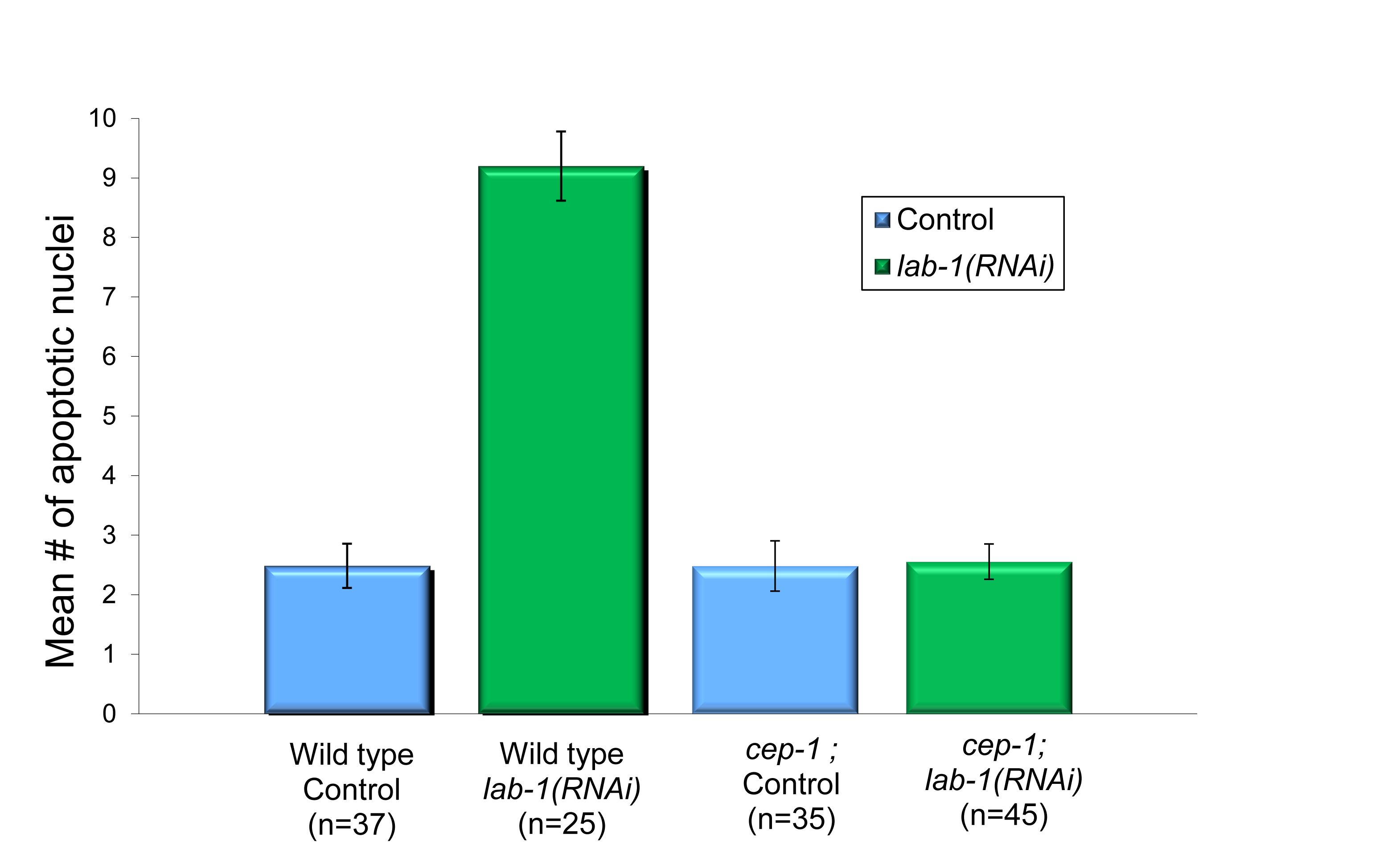

Supplement: Figure S4 — Increased germ cell apoptosis in lab-1(RNAi) worms is CEP-1/p53-dependent, indicating activation of a DNA damage checkpoint. Quantification of germline apoptosis by scoring acridine orange positive nuclei in control, cep-1 control, lab-1(RNAi), and cep-1 lab-1(RNAi) worms. Error bars represent standard deviation of the mean. n, number of gonad arms scored. (TIF) [file pbio.1001378.s004.tif]

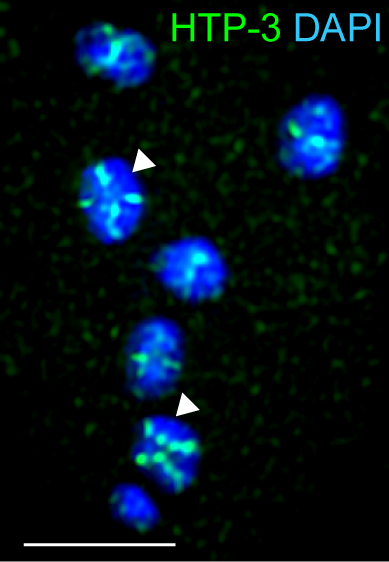

Supplement: Figure S5 — Detection of chiasmata in lab-1-depleted gonads. Partial projection of a z stack of images collected from a diakinesis nucleus in a lab-1(RNAi) gonad co-stained with HTP-3 (green) and DAPI (blue). Arrowheads point towards bivalents in which a single chiasma is clearly detected by the cruciform organization of the axes highlighted by HTP-3. Bar, 4 µM. (TIF) [file pbio.1001378.s005.tif]

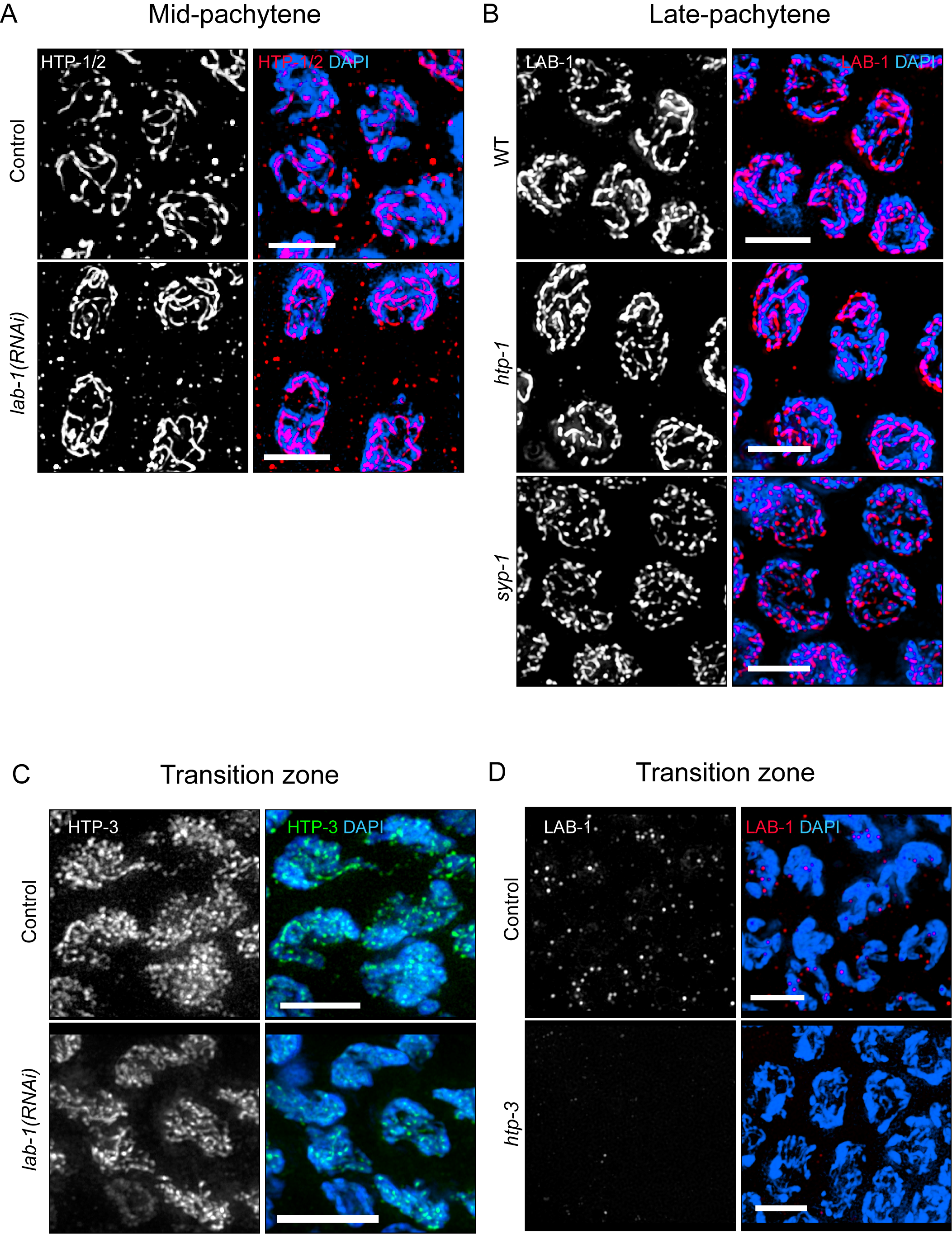

Supplement: Figure S6 — Interdependency analysis of chromosomal localization of the HTP-1/-2/-3 and LAB-1 proteins. (A) Mid-pachytene nuclei in control and lab-1(RNAi) gonads co-stained with HTP-1/2 (red) and DAPI (blue). (B) Late-pachytene nuclei in wild type, htp-1, and syp-1 mutants co-stained with LAB-1 (red) and DAPI (blue). (C) Transition zone nuclei in control and lab-1(RNAi) gonads co-stained with HTP-3 (green) and DAPI (blue). (D) Transition zone nuclei in wild type, and htp-3 mutants co-stained with LAB-1 (red) and DAPI (blue). Bars, 4 µM. (TIF) [file pbio.1001378.s006.tif]

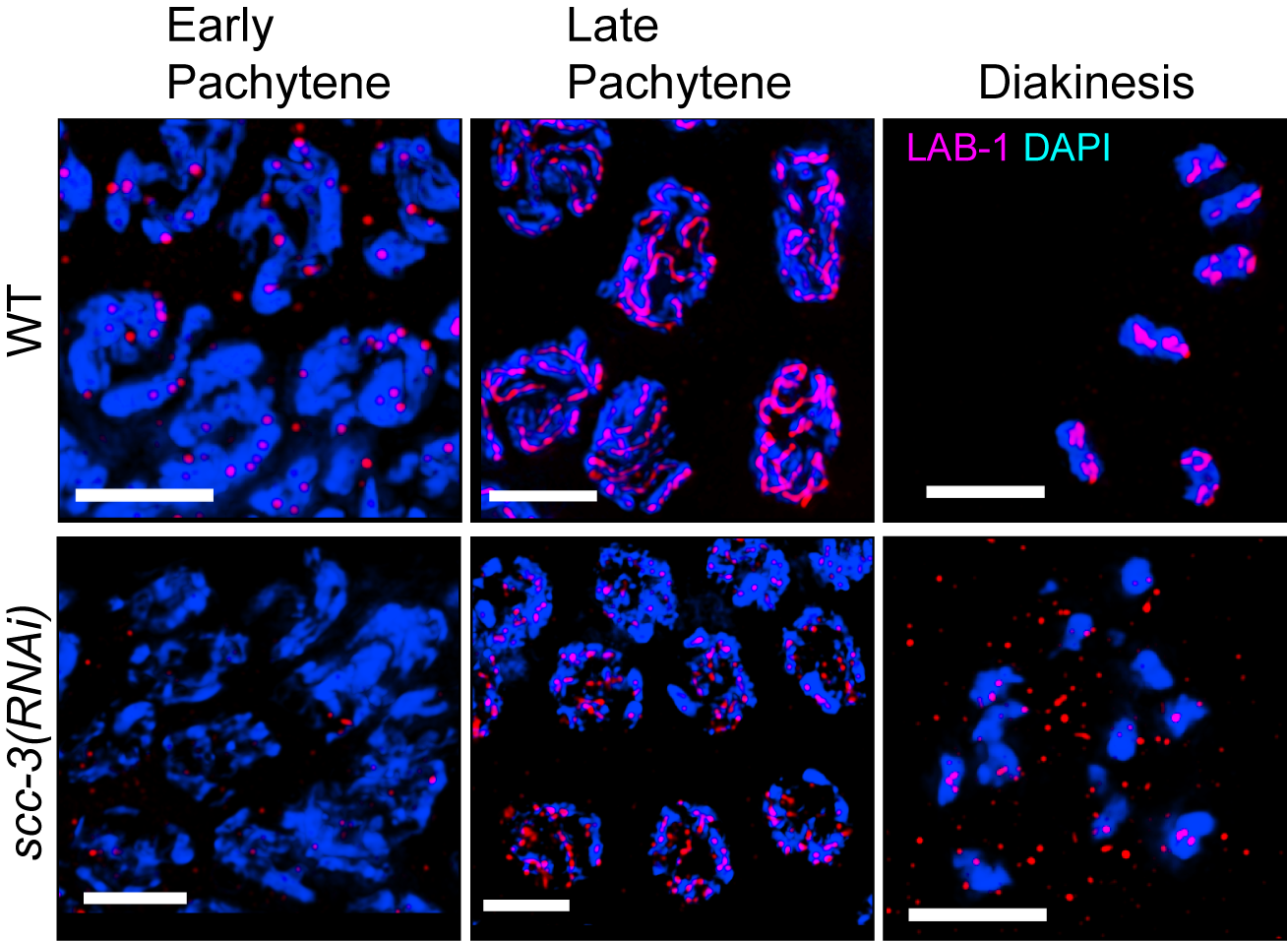

Supplement: Figure S7 — LAB-1 localization is SCC-3-dependent. High-magnification images of early pachytene and late pachytene nuclei as well as −1 oocytes at diakinesis co-stained with LAB-1 (red) and DAPI (blue) in scc-3(RNAi) gonads. Bars, 4 µM. (TIF) [file pbio.1001378.s007.tif]

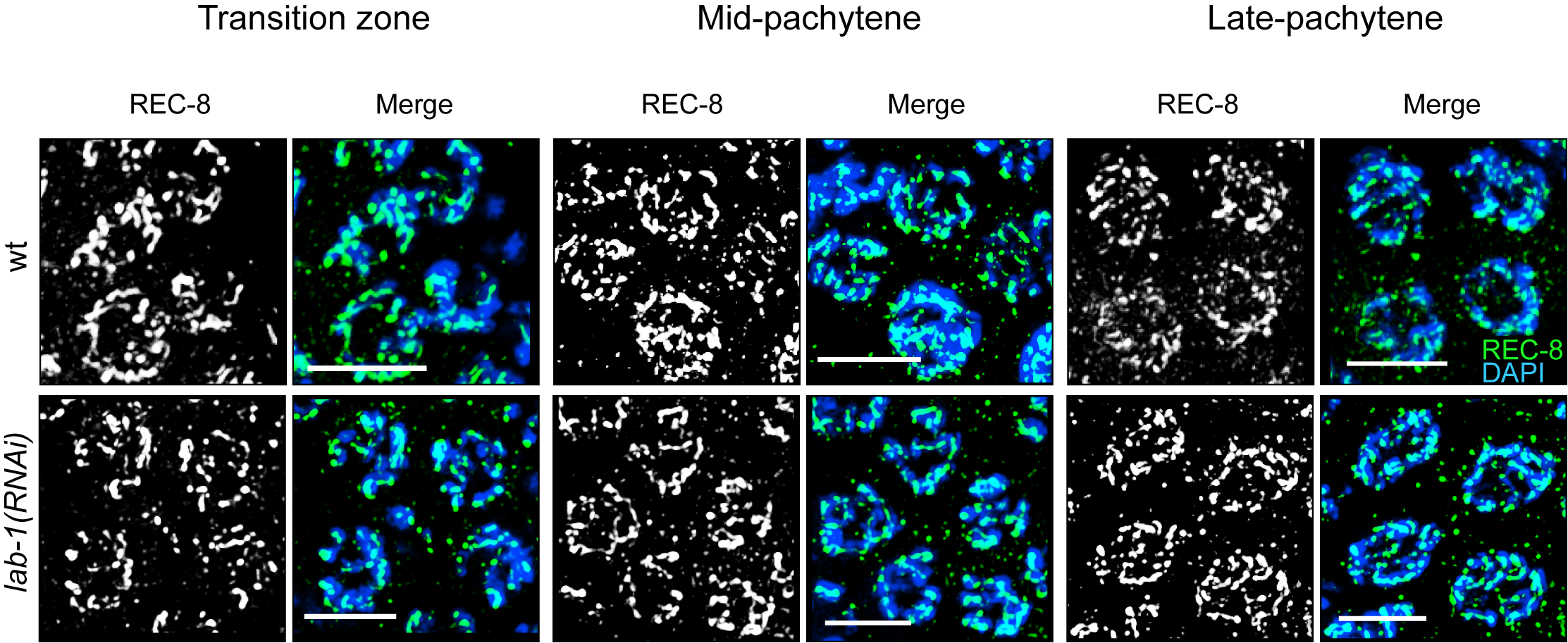

Supplement: Figure S8 — REC-8 localization in early prophase I is not altered following lab-1 depletion. High-magnification images of transition zone, mid-pachytene, and late pachytene nuclei co-stained with REC-8 (green) and DAPI (blue) in control and lab-1(RNAi) germlines. Bars, 4 µM. (TIF) [file pbio.1001378.s008.tif]

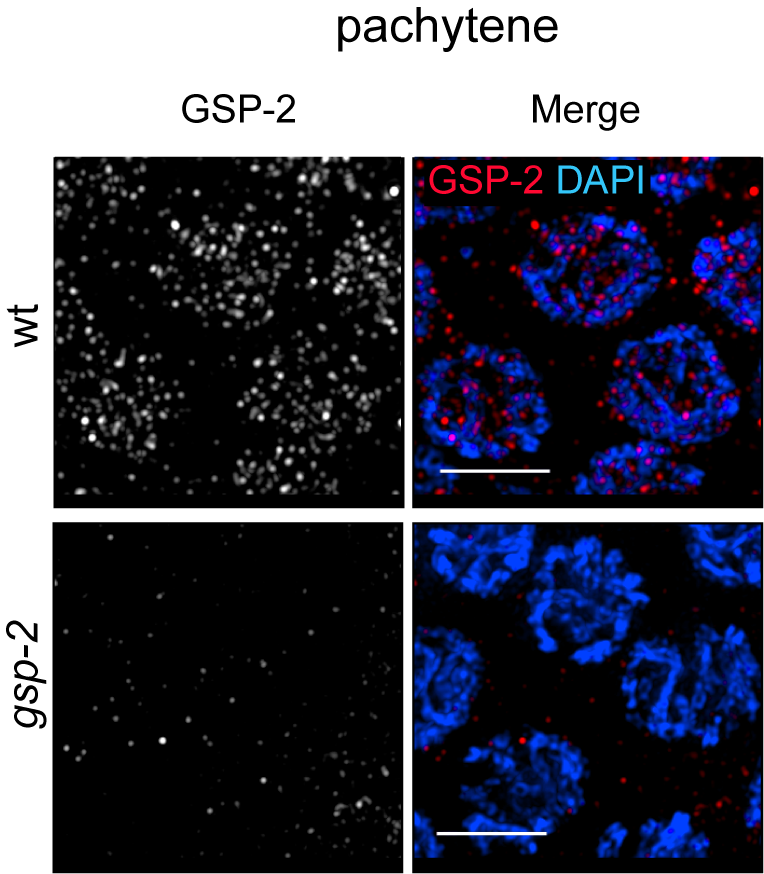

Supplement: Figure S9 — Specificity of GSP-2 antibodies. High-magnification images of pachytene nuclei co-stained with GSP-2 (red) and DAPI (blue) in wild-type and gsp-2 germlines. Bars, 4 µM. (TIF) [file pbio.1001378.s009.tif]

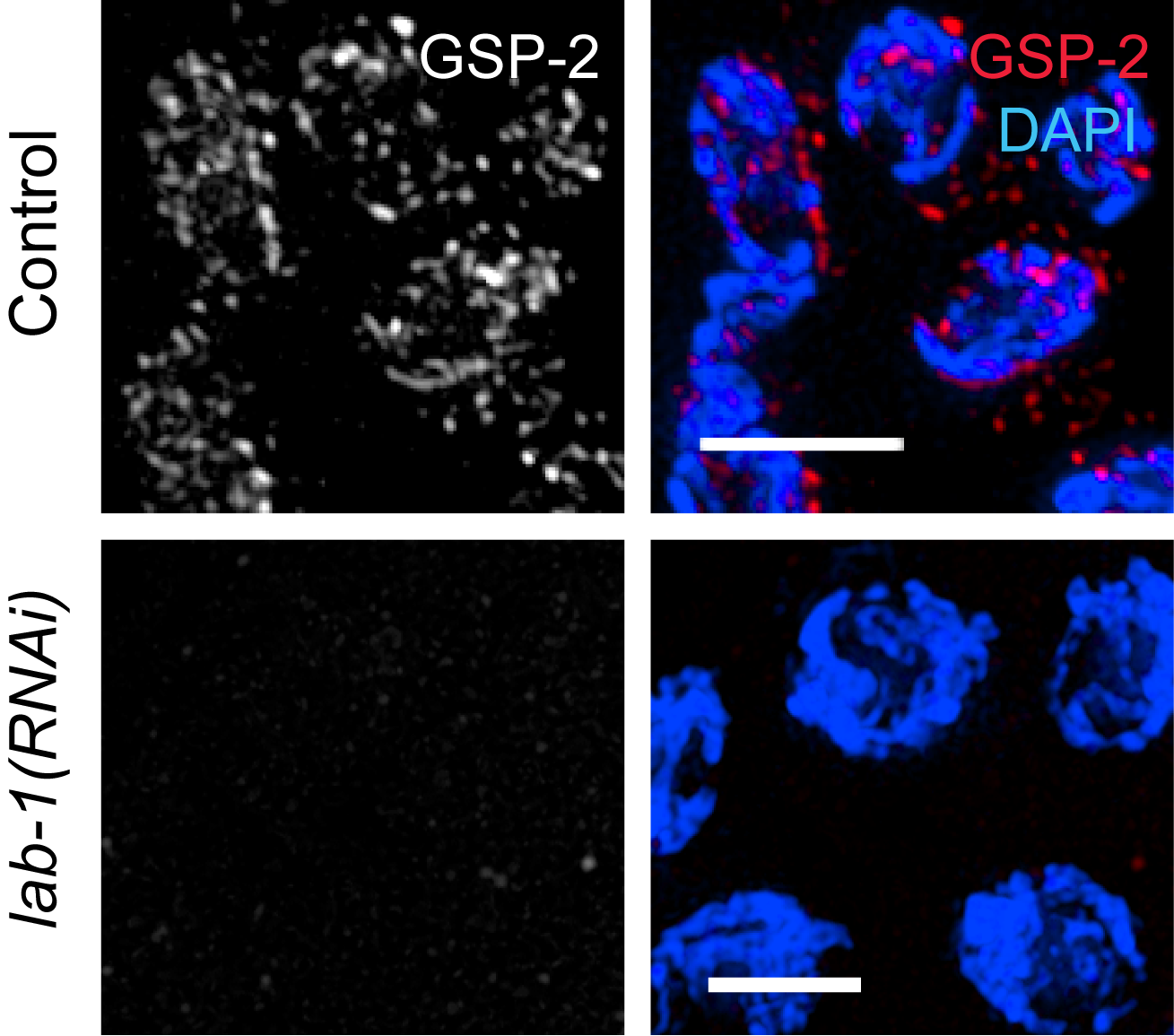

Supplement: Figure S10 — GSP-2 signal associated with transition zone nuclei is LAB-1-dependent. Transition zone nuclei in control and lab-1(RNAi) gonads mildly squashed as in [83], and co-stained with GSP-2 (red) and DAPI (blue). Bars, 4 µm. (TIF) [file pbio.1001378.s010.tif]

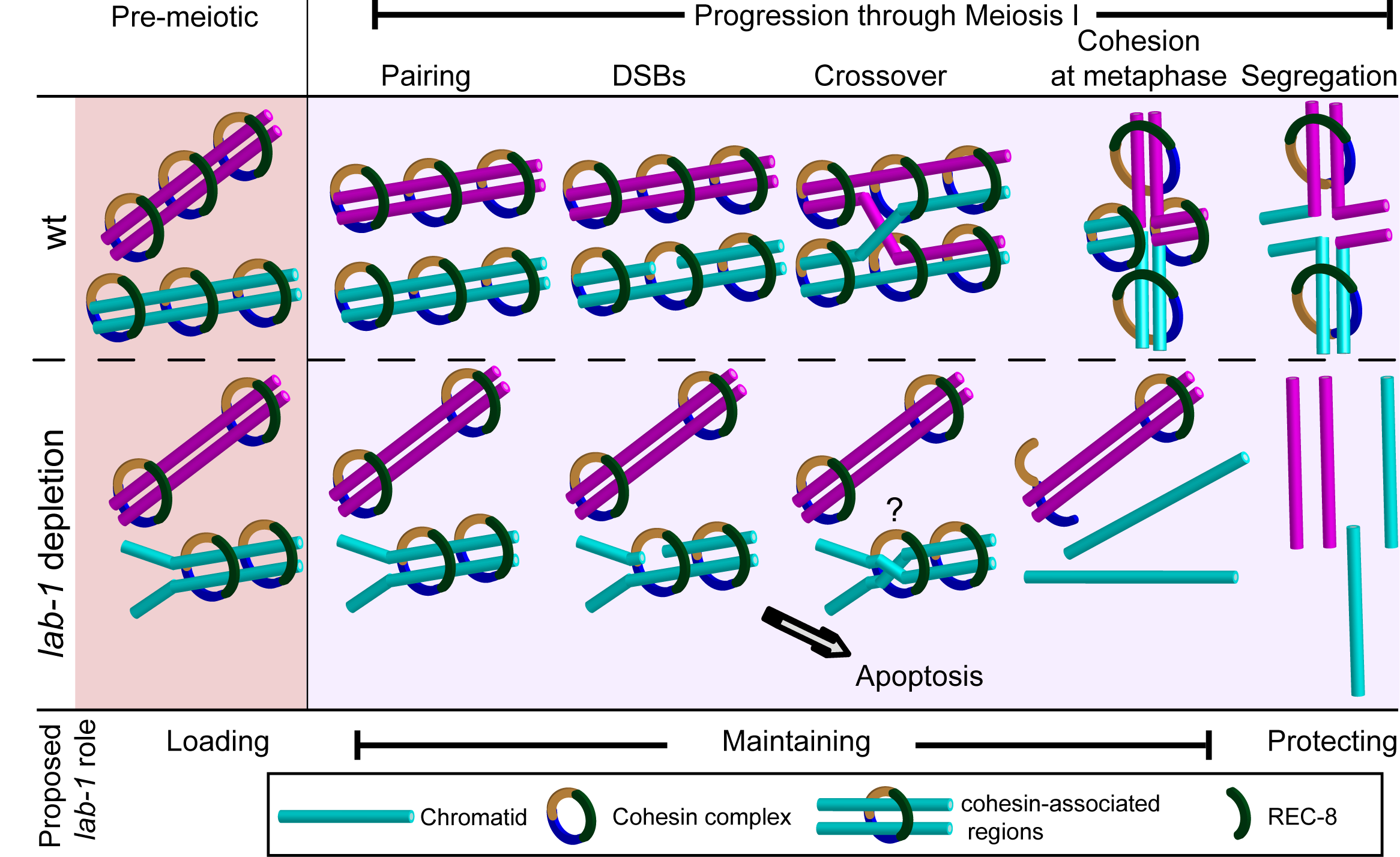

Supplement: Figure S11 — Depletion of lab-1 SCC reduction manifests as meiotic defects. We propose that LAB-1 assists in cohesin loading in the pre-meiotic region, and maintenance of the complex during early prophase I, whereas it protects REC-8 from premature removal at the long arms of the bivalents at metaphase I. When lab-1 is depleted, cohesin is not loaded correctly, potentially creating localized regions with either a lack or reduction of cohesin. The partial dissociation of sister chromatids reduces homologous pairing and impairs the repair of DSBs via interhomolog recombination, possibly due to the lack of a stable homologous template in close proximity. This results in either apoptosis or the use of alternative modes of meiotic DSB repair, such as intersister-based repair. Upon remodeling, the lack of both SCC and interhomolog crossovers leads to the formation of both univalents and single chromatids. Lack of LAB-1 in metaphase I results in the premature removal of REC-8 from the long arms and increased errors in chromosome segregation. (TIF) [file pbio.1001378.s011.tif]

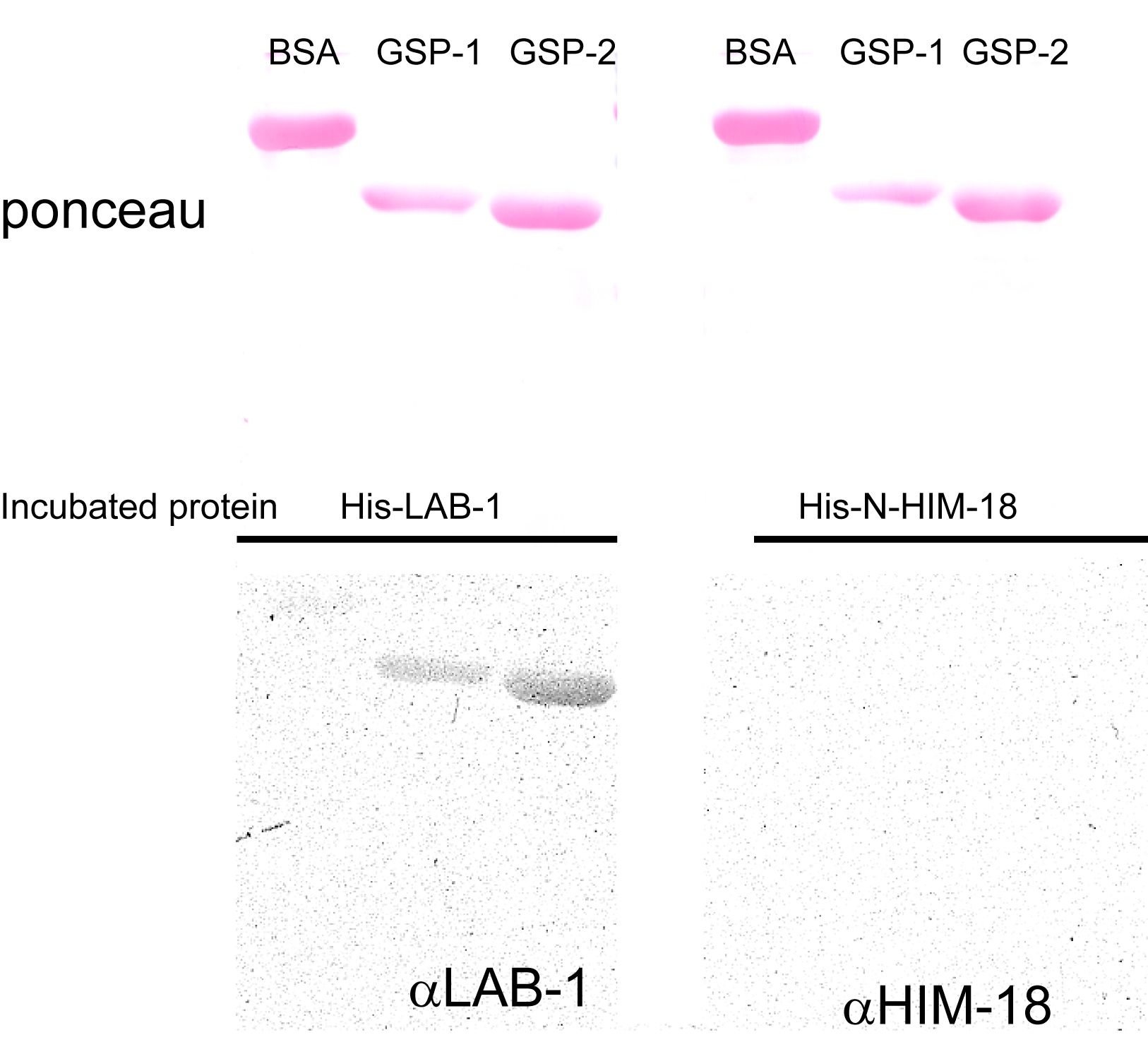

Supplement: Figure S12 — LAB-1 can specifically bind GSP-1 and GSP-2 in vitro. Far-western assay for in vitro binding of purified recombinant LAB-1 and N-HIM-18 (negative control) to GSP-1 and GSP-2 transferred to membranes. (TIF) [file pbio.1001378.s012.tif]
