# Supplementary material for: LAB-1 Targets PP1 and Restricts Aurora B Kinase upon Entrance into Meiosis to Promote Sister Chromatid Cohesion
Source: PLoS Biol. 2012 Aug 21;10(8):e1001378. doi: 10.1371/journal.pbio.1001378 (PMC3424243; doi:10.1371/journal.pbio.1001378)
Supplement: Table S1 — LAB-1 interacting proteins. Immunoprecipitation (IP) from LAB-1::GFP whole worm extracts with an antibody against GFP was analyzed by mass spectrometry. Numbers indicate the total mass spectra collected in two samples. (DOC) [file pbio.1001378.s013.doc]

Table S1:

| Protein ID | Protein name/Description | Predicted Molecular Weight (Da) | Sequence Coverage (%) |
| --- | --- | --- | --- |
| CE07625 | HIM-3 | 33121 | 56.4 |
|  | GFP | 26813 | 47.5 |
| CE31004 | Confirmed UniProt:Q8WQE8 | 28598 | 46.3 |
| CE31692 | HTP-1 | 39619 | 43.2 |
| CE37734 | HTP-2 | 39614 | 38.9 |
| CE13096 | **Confirmed UniProt:P91430** | 46512 | 31.7 |
| CE29377 | HMG-1.1 | 10613 | 28.4 |
| CE33885 | Partially_confirmed UniProt:Q95XX1 | 61309 | 25.4 |
| CE33886 | Partially_confirmed UniProt:Q95XX1 | 63446 | 24.5 |
| CE00015 | **DARS-1** | 59939 | 18.1 |
| CE11330 | HTP-3 | 81566 | 15.8 |
| CE17464 | Confirmed UniProt:O76387 | 33689 | 14.6 |
| CE06020 | **Confirmed UniProt:Q20981** | 32684 | 13.8 |
| CE15580 | LAB-1 | 18577 | 13.7 |
| CE28902 | GLNA-2 | 68336 | 12.7 |
| CE14556 | Partially_confirmed UniProt:O44749 | 59138 | 12.2 |
| CE23949 | Confirmed UniProt:O18034 | 40968 | 9.8 |
| CE36335 | CRTC-1 | 51683 | 5.7 |
| CE03073 | Partially_confirmed UniProt:Q18494 | 101231 | 4.7 |
| CE42000 | Partially_confirmed UniProt:A9UJN7 | 91979 | 5.1 |
| CE09632 | Partially_confirmed UniProt:Q93562 | 98521 | 4.8 |
|  |  |  |  |
|  |  |  |  |

Immunoprecipitation (IP) from LAB-1::GFP whole worm extracts with an antibody against GFP was analyzed by mass spectrometry. Numbers indicate the total mass spectra collected in 2 samples.
